# New Quinoxaline Derivatives as Dual Pim-1/2 Kinase Inhibitors: Design, Synthesis and Biological Evaluation

**DOI:** 10.3390/molecules26040867

**Published:** 2021-02-06

**Authors:** Bruno Oyallon, Marie Brachet-Botineau, Cédric Logé, Thomas Robert, Stéphane Bach, Sajida Ibrahim, William Raoul, Cécile Croix, Pascal Berthelot, Jean Guillon, Noël Pinaud, Fabrice Gouilleux, Marie-Claude Viaud-Massuard, Caroline Denevault-Sabourin

**Affiliations:** 1 EA GICC-ERL 7001 CNRS, Team IMT, University of Tours, F-37200 Tours, France; bruno.oyallon@gmail.com (B.O.); cecile.croix@univ-tours.fr (C.C.); marie-claude.viaud-massuard@univ-tours.fr (M.-C.V.-M.); 2 CNRS ERL7001 LNOx-EA GICC, University of Tours, F-37000 Tours, France; marie.brachet.botineau@gmail.com (M.B.-B.); fabrice.gouilleux@univ-tours.fr (F.G.); 3 Department of Medicinal Chemistry, IICIMED-EA1155, IRS2, University of Nantes, F-44200 Nantes, France; cedric.loge@univ-nantes.fr; 4 Integrative Biology of Marine Models Laboratory (LBI2M), Sorbonne University, CNRS, UMR8227, F-29680 Roscoff, France; trobert@sb-roscoff.fr (T.R.); bach@sb-roscoff.fr (S.B.); 5 Kinase Inhibitor Specialized Screening facility (KISSf), Sorbonne University, CNRS, FR2424, F-29680 Roscoff, France; 6 Centre of Excellence for Pharmaceutical Sciences, North-West University, Private Bag X6001, Potchefstroom 2520, South Africa; 7 EA GICC-ERL 7001 CNRS, Team PATCH, University of Tours, F-37200 Tours, France; sajidaibrahim@hotmail.com (S.I.); william.raoul@univ-tours.fr (W.R.); 8 N2C, University of Tours, INSERM, UMR 1069, F-37032 Tours, France; 9 UMR-S 1172-JPArc, University of Lille, INSERM, CHU Lille, F-59000 Lille, France; pascal.berthelot@univ-lille2.fr; 10 ARNA Laboratory, University of Bordeaux, INSERM U12132-UMR CNRS 5320, F-33076 Bordeaux, France; jean.guillon@u-bordeaux.fr; 11 ISM, University of Bordeaux, CNRS, UMR 5255, F-33405 Talence, France; noel.pinaud@u-bordeaux.fr

**Keywords:** quinoxaline, Pim kinases, kinase inhibitor, anticancer targeted therapy

## Abstract

Proviral integration site for Moloney murine leukemia virus (Pim)-1/2 kinase overexpression has been identified in a variety of hematologic (e.g., multiple myeloma or acute myeloid leukemia (AML)) and solid (e.g., colorectal carcinoma) tumors, playing a key role in cancer progression, metastasis, and drug resistance, and is linked to poor prognosis. These kinases are thus considered interesting targets in oncology. We report herein the design, synthesis, structure–activity relationships (SAR) and in vitro evaluations of new quinoxaline derivatives, acting as dual Pim1/2 inhibitors. Two lead compounds (**5c** and **5e**) were then identified, as potent submicromolar Pim-1 and Pim-2 inhibitors. These molecules were also able to inhibit the growth of the two human cell lines, MV4-11 (AML) and HCT-116 (colorectal carcinoma), expressing high endogenous levels of Pim-1/2 kinases.

## 1. Introduction

Proviral integration site for Moloney murine leukemia virus (Pim) kinases 1, 2 and 3 are homologous constitutively active proto-oncogenic serine/threonine protein kinases, which share a high level of sequence homology, and subsequent functional redundancies [1,2]. Nevertheless, they differ partially in their tissue distribution. Pim-1 and Pim-2 are mainly expressed in hematopoietic cells, while Pim-3 is more abundant in breast, brain, kidneys, and epithelia [3]. These oncoproteins are positive regulators of cell cycle progression, and inhibit apoptosis, acting as oncogenic survival factors [4,5]. They play a critical role in the control of cell proliferation, survival, differentiation, and migration [6,7]. Pim kinases are aberrantly up-regulated in a variety of hematologic (e.g., multiple myeloma, acute and chronic myeloid leukemias) and solid (e.g., prostate, breast, colon) tumors [8,9,10,11,12], contributing to malignant transformation, cancer progression, metastasis, drug resistance and often poor prognosis. The Pim kinases overexpression observed in cancer cells results from the abnormal activation of upstream kinases (e.g., BCR-Abl, Jak2) or receptor tyrosine kinases (RTK) (e.g., fms-like tyrosine kinase 3 (FLT3)-ITD), responsible for the activation of the signal transducer and activator of transcription (STAT) family transcription factors (particularly STAT 3/5) [13].

Interestingly, it has been demonstrated that Pim kinases are involved in the expression, activation, and stabilization of drug efflux transporters, contributing to multidrug resistance. More specifically, Pim-1 phosphorylates the P-glycoprotein (Pgp), resulting in its protection from ubiquitination and proteasomal degradation. Pim-1 also mediates phosphorylation of the breast-cancer-resistant protein (BCRP), promoting its multimerization and stable membrane expression [14,15]. Consequently, Pim kinases, by promoting survival signals, and multiple drug resistance can reduce the efficacy of chemotherapeutic drugs.

All these findings, and the fact that *pim* genes triple knockout mice demonstrated mild phenotypic changes [16], highlight the therapeutic potential of these kinases as targets in oncology, especially in drug-resistant tumors, in order to restore chemosensitivity to classical chemotherapies. Moreover, because of the redundant functions of the three Pim isoforms, there is currently a great interest for the development of pan-Pim inhibitors.

**Figure 1:**
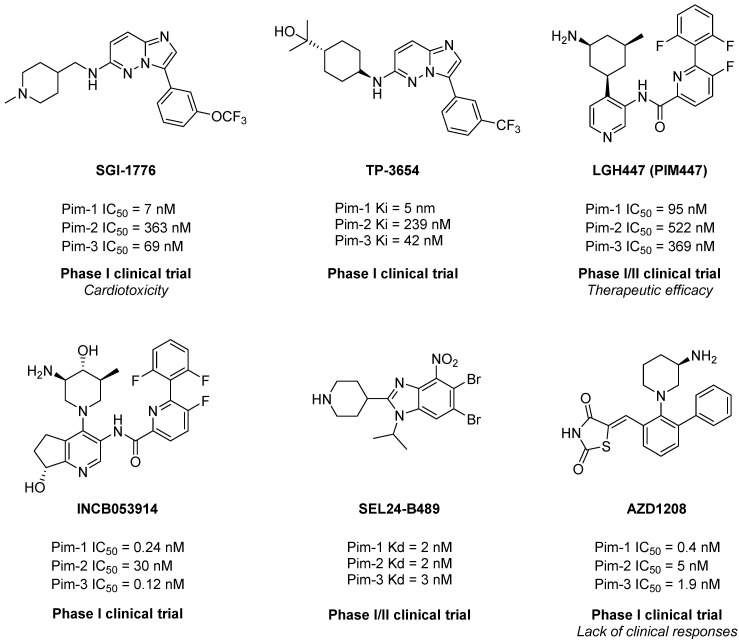
Selection of the most representative and/or promising proviral integration site for Moloney murine leukemia virus (Pim) inhibitors.

A number of different chemical classes of Pim inhibitors have been reported to date, including, for example, imidazo [1,2-*b*]pyridazines, thiazolidine-2,4-diones, benzo[*d*]imidazoles or pyridines [17,18] (Figure 1). For the most promising candidates, clinical trials were carried out or are currently ongoing [18,19,20,21]. Thus, the pan-PIM inhibitors PIM447 (LGH447, Novartis) demonstrated antitumor activity in monotherapy in patients with relapsed/refractory multiple myeloma with a good tolerance profile [21].

Crystallographic studies of Pim-1 and Pim-2 revealed unique structural features of Pim kinases. Thus, in all three Pim kinases, the hinge region is characterized both by an atypical conformation and by the lack of a hydrogen bond (H-bond) donor in the hinge. Indeed, this region contains two proline residues (Pro123 and Pro125, in Pim-1), with no H-bond donor property. Consequently, Pim kinases bound ATP, the natural substrate, via only one hinge H-bond, involving the ATP adenine amino substituent and the hinge glutamate (Glu121, in Pim-1) backbone carbonyl. Moreover, the lateral chain of the hinge prolines and other hydrophobic amino acid residues in their environment, create a hydrophobic pocket in the hinge region. Such differences between Pim kinases and other kinases provide an opportunity for the design of selective pan-Pim inhibitors [22,23].

As part of our laboratory drug discovery program aimed at identifying new targeted therapies for the treatment of hematologic malignancies, and, regarding the particular potential of Pim-1 and Pim-2 as targets in leukemia [24,25], we decided to develop new dual Pim-1/Pim-2 specific inhibitors.

**Figure 2:**
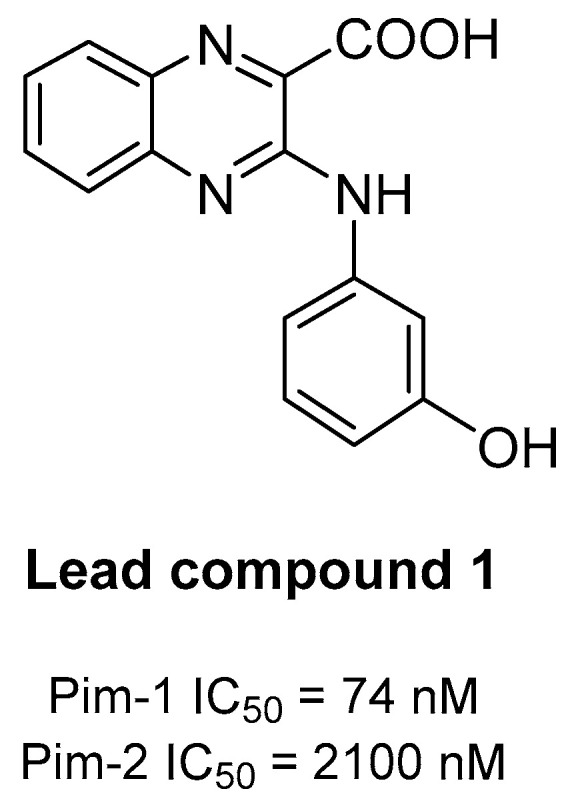
Chemical structure of lead compound **1** [26].

In a previous work, we identified the quinoxaline-2-carboxylic acid **1** as a new lead compound (Figure 2). This molecule potently inhibits Pim-1 enzymatic activity at submicromolar concentrations (IC_50_ of 74 nM) [26]. However, recent studies showed that this compound exerted only a modest activity against Pim-2 with an IC_50_ of 2.10 µM (data not published). In the light of these results, we planned to prepare analogues of compound **1**, with an optimized activity on both Pim-1 and Pim-2 isoforms.

Pim inhibitors of the literature can be classified into two classes: ATP mimetics, which establish an H-bond with the hinge glutamate residue (Glu121, in Pim-1), and non-ATP mimetics, which bind distant to the hinge or interact with the hinge through weak (mainly hydrophobic) interactions, with multiple residues of the unique hydrophobic pocket in the hinge environment. It has been shown that non-ATP mimetic Pim inhibitors could achieve higher selectivity over other kinases [27].

Consequently, in this work, we decided to probe the unique hydrophobic pocket of the Pim kinases hinge environment, by adding halogenated substituents in positions 6 or 7 of the quinoxaline scaffold, oriented towards the hinge region of the ATP binding site, and able to form weak interactions in this area. Eight new derivatives were then prepared and tested for their Pim-1 and Pim-2 inhibitory activity. The selectivity profiles and anti-proliferative activities of the most active compounds were further evaluated against a panel of mammalian kinases and against four different cell lines, respectively.

## 2. Results and Discussion

### 2.1. Synthesis

**Scheme 1:**
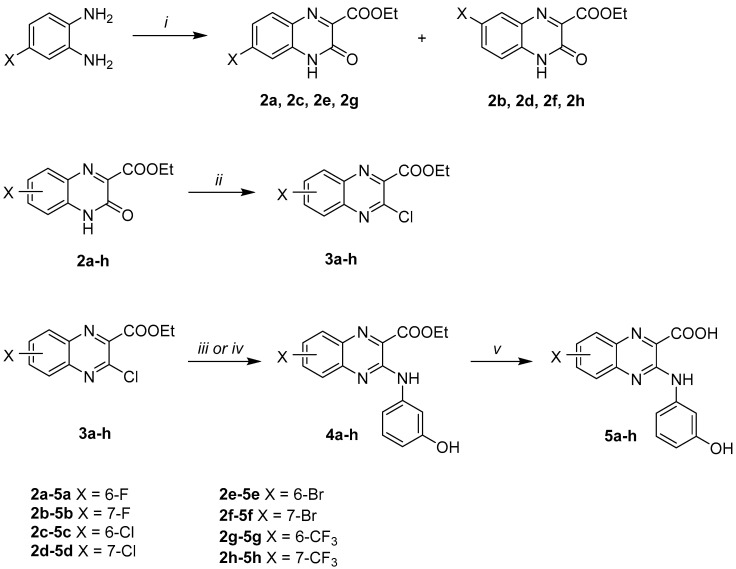
Reagents and conditions: (*i*) diethyl 2-oxomalonate (1 eq), citric acid (15 mol%), EtOH, rt, 2–24 h, 16–54%; (*ii*) *N*,*N*-dimethylformamide (DMF) (cat.), POCl_3_, 0 °C, and then reflux, 3 h, 51–100%; (*iii*) 3-aminophenol (1.1–3 eq), EtOH, MW 150 °C, 3 h, 29–99%; (*iv*) 3-aminophenol (3 eq), p-toluenesulfonic acid (cat.), EtOH, MW 100 °C, 8 h, 70%; (*v*) K_2_CO_3_ (3 eq), MeOH/H2O (4/1), reflux, 4 h, 94–100%.

The preparation of the 6- or 7-substituted-quinoxaline-2-carboxylic acids was performed as described in Scheme 1, from commercial *o*-phenylenediamines according to literature procedures [28,29].

First, the appropriate 4-substituted-1,2-phenylenediamine was condensed with diethyl 2-oxomalonate in the presence of citric acid (15 mol%) at room temperature in ethanol to give, after separation by column chromatography, ethyl 6-substituted-3-oxo-3,4-dihydroquinoxaline-2-carboxylates **2a**, **2c**, **2e**, and **2g**, and their seven-substituted isomers **2b**, **2d**, **2f**, and **2h**, respectively. The identification of each isomer was realized by comparison with literature spectroscopic data [30,31].

Subsequent chlorination in position 3 of compounds **2a–h** using *N*,*N*-dimethylformamide (DMF) as a catalyst in refluxing phosphorous oxychloride afforded intermediates **3a–h**, which were used directly for the next step because of a lack of stability.

**Figure 3:**
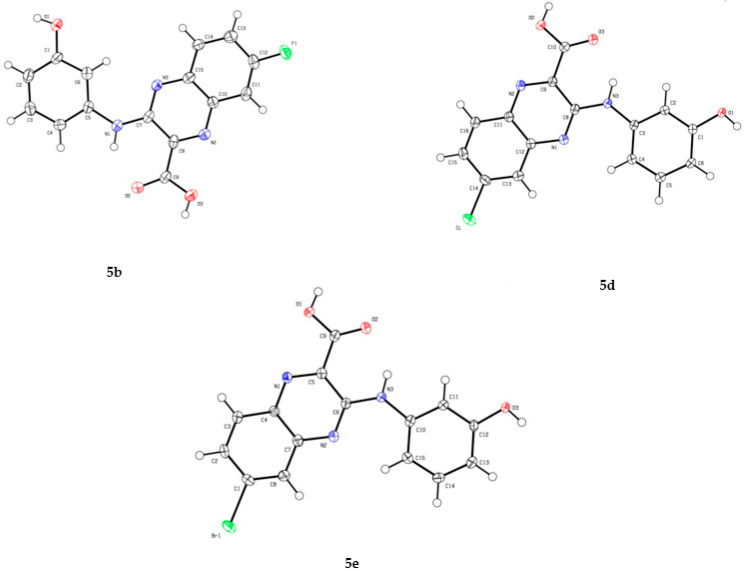
Oak Ridge Thermal-Ellipsoid Plot Program (ORTEP) drawing of substituted-quinoxaline-2-carboxylic acids **5b**, **5d** and **5e** with thermal ellipsoids at 30% level [32].

Access to quinoxaline-2-carboxylic acids was then performed using a two-step synthetic pathway. Intermediates **3a–h** undergo initial nucleophilic aromatic substitution with 3-aminophenol in ethanol under microwave irradiation, to yield esters **4a–h**. For compound **4g** synthesis, a catalytic amount of *p*-toluenesulfonic acid (*p*-TSA) was used. Then, hydrolysis of intermediate ethyl esters **4a–h** with potassium carbonate in refluxing 80% aqueous methanol was performed, followed by an acidification with a 15% hydrochloric acid aqueous solution to afford acids **5a–h**. The 3D structural determination of the substituted-quinoxaline-2-carboxylic acids **5b**, **5d** and **5e** was established by X-ray crystallography (Figure 3) [32] and confirmed the structure in the solid state.

### 2.2. Enzymatic Assays

#### 2.2.1. Pim-1 and Pim-2 Enzymatic Activity Inhibition

Synthesized compounds were first evaluated for their ability to inhibit the in vitro enzymatic activity of *Homo sapiens* Pim-1 (*Hs*Pim-1) and *Hs*Pim-2, using a luminescence-based kinase assay [33]. SGI-1776, a small-molecule pan-Pim protein kinase inhibitor [18], commercially available, was used as a control for the in vitro studies. To increase our inhibitor affinity on Pim kinases, we first decided to structurally vary the substitution patterns of the quinoxaline scaffold of **1** in positions 6 and 7, introducing halogenated substituents, trying to establish hydrophobic interactions with the unique hinge hydrophobic pocket (Table 1).

In position 7, it appears that the nature of the halogenated group has important repercussions on compounds activity. Thus, introduction, in this position, of a bromine led to the potent derivative **5f**. This compound maintained a submicromolar activity on Pim-1 (IC_50_ of 180 nM) with approximately the same level of activity than lead compound **1** on both Pim isoforms (Table 1, entries 1 and 7). In contrast, the 7-substitution by a fluorine or a chlorine atom or by a trifluoromethyl group was not favorable for the activity, as evidenced by derivatives **5b**, **5d** and **5h** (IC_50_ > 1 µM on both Pim-1 and Pim-2 isoforms) (Table 1, entries 3, 5 and 9).

In position 6, similar Pim-1 inhibition trends were observed with all halogenated substituents whatever their size (Table 1, entries 2, 4, 6 and 8), with IC_50_ values in the range of 130 to 200 nM, in every case, and nearly the same level of potency than lead compound **1** (Table 1, entry 1). Interestingly, the inhibition profile on Pim-2 isoform was significantly improved for smaller **5a** (6-F) and bulkier **5e** (6-Br) derivatives, with IC_50_ values 3- and 4-fold lower than lead **1**, respectively. Finally, the most active compound **5c** (6-Cl) was 12-fold more active on Pim-2 isoform than lead **1**, and exhibited the same level of activity than the reference drug SGI-1776 on both isoforms (Table 1, entry 10).

#### 2.2.2. Selectivity over a Panel of Mammalian Protein Kinases

To compare the selectivity profile of our inhibitors, most active candidates were further evaluated against a selected panel of mammalian protein kinases (comprising *Rn*DYRK1A, *Hs*CDK5/p25, *Hs*CDK9/CyclinT, *Hs*Haspin, *Mm*CLK1, *Hs*CK1ε and *Hs*GSK3β) using a luminescence-based kinase assay [33].

As it can be observed with lead compound **1**, derivatives **5c** and **5e** maintained an interesting selectivity profile against the five potential off-target kinases *Hs*CDK5/p25, *Hs*CDK9/CyclinT, *Hs*Haspin, *Mm*CLK1 and *Hs*CK1ε with IC_50_ inhibition values > 10 µM in every case (Table 2, entries 1, 2 and 3).

The new quinoxalines tested have limited activity against *Hs*GSK3β, displaying micromolar inhibition, in contrast to lead **1** (IC_50_ > 10 µM) (Table 2, entries 1, 2 and 3). Our best inhibitor **5c** exhibited nevertheless an IC_50_ value at least 21- and 16-fold higher for *Hs*GSK3β than for *Hs*Pim-1 and *Hs*Pim-2, respectively.

In contrast, while compound **1** potently inhibits *Rn*DYRK1A (IC_50_ of 0.27 µM), the most potent quinoxalines, **5c** and **5e**, displayed only micromolar inhibition of *Rn*DYRK1A (Table 2, entries 1, 2 and 3).

Importantly, the general selectivity profile of our best inhibitors (**5c** and **5e**) was significantly improved in comparison to the reference drug SGI-1776, which inhibited eight of the nine mammalian kinases tested, with IC_50_ values in the submicromolar to low micromolar range (0.05–9.53 µM) (Table 2, entry 4).

### 2.3. Docking Studies

**Figure 4:**
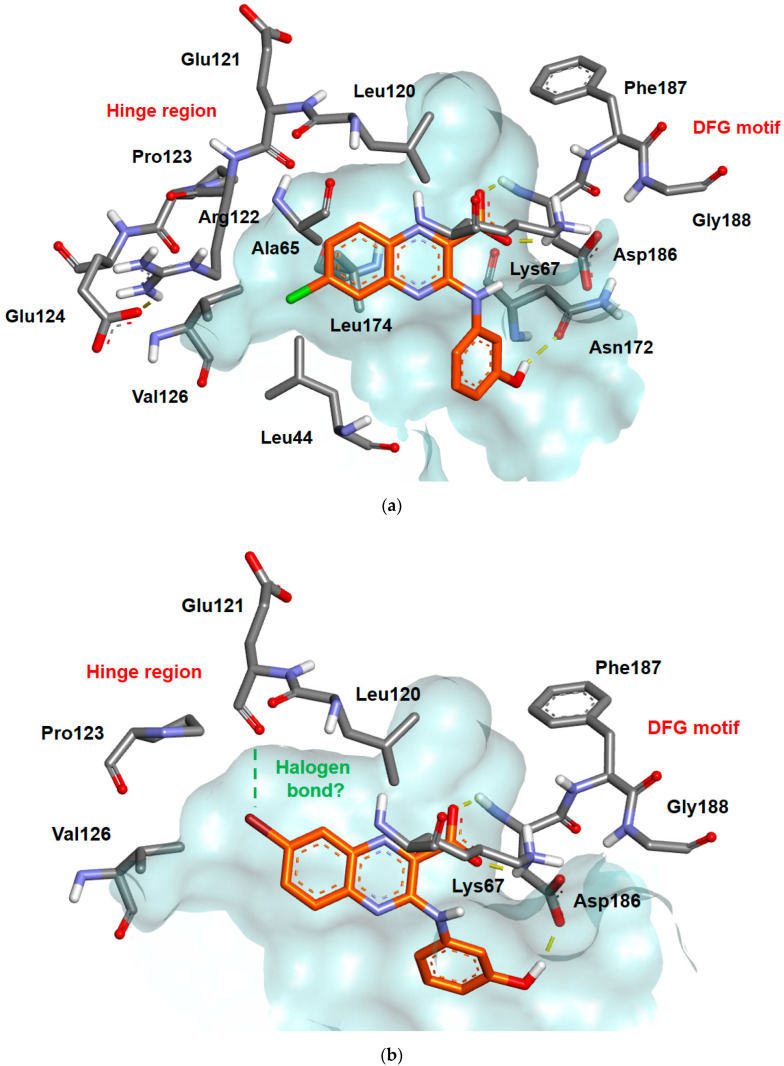
(**a**) Illustration of the possible binding mode within the ATP pocket of Pim-1 of compound **5c** (**b**) Illustration of the possible binding mode within the ATP pocket of Pim-1 of compound **5f**. Hydrogen bonds are indicated as yellow lines. Pim-1 PDB ID: 3A99.

As already hypothesized for compound **1**, docking analysis of compound **5c** (6-Cl) into the ATP pocket of Pim-1 (Figure 4a) showed a key salt bridge between the carboxylate function in position 2 of the quinoxaline scaffold and the ammonium side chain of the catalytically essential Lys67. As expected, the 6-chloro substituent is oriented towards the hydrophobic pocket formed by Leu44 (P-loop residue), Ala65, the alkyl side chain of Arg122, Val126 and Leu174 and contributes to enhance van der Waals interactions. This hypothesis can be extrapolated for the other 6-halogenated substituents (**5a** (6-F), **5e** (6-Br)) and for the 6-trifluoromethyl group (**5g**). Isomerization of the bromine atom from the 6- to the 7-position (**5f**) does not modify the IC_50_ values in Pim-1, while it impacts ones with a fluorine atom (**5b**), chlorine atom (**5d**) or a trifluoromethyl group (**5h**). The docking results of compound **5f** (Figure 4b) into the ATP pocket of Pim-1 revealed a similar salt bridge interaction with Lys67, even if the 7-bromo substituent can influence both conformation and binding in this sterically restricted area of the hinge region, probably to promote a halogen-bond interaction with the carbonyl group of Glu121. This hypothesis is consistent with the halogen-bonding theory where heavier halogens bound to aryl groups show a partial positive charge opposite the C-X bond (with the exception of fluorine atoms due to their high electronegativity) that allows for interactions with classical H-bond acceptors [34].

### 2.4. In Vitro Cell-Based Assays

Most active Pim inhibitors were then tested in vitro on the human acute myeloid leukemia (AML) cell line MV4-11, harboring the FLT3-ITD mutation, and overexpressing Pim kinases. Indeed, Pim 1 and Pim-2 kinases have been shown to be key downstream effectors of FLT3-induced signaling [35,36]. Moreover, the FLT3-ITD mutation confers poor prognosis in patients with AML [36]. Cytotoxic effects were evaluated using an MTT assay, and living cells were also counted with the trypan blue dye exclusion method (Table 3).

The most potent Pim1/2 inhibitors, **5c** and **5e**, exhibited in vitro cytotoxic effects on the MV4-11 cell line with EC_50_ values in the micromolar range (35.5 ± 1.1 µm and 32.9 ± 9.6 µM, respectively), and a better activity than the lead compound **1** (EC_50_ of 61.2 ± 3.9 µM) (Table 3, entries1, 2 and 3).

To determine if the growth inhibitory activity of these compounds was restricted to leukemic cells, we analyzed the effects of these quinoxalines on normal bone marrow stromal and mesenchymal stem cells, major cellular components of the leukemic and hematopoietic microenvironment. To this purpose, we used bone marrow mesenchymal stem cells (MSCs), freshly isolated from brain-dead donors, and the human bone marrow stromal cell line HS-27a, sharing similar biological properties with MSCs.

The results show that, as can be observed with lead compound **1**, quinoxaline **5c** demonstrated a moderate inhibitory activity on these cells’ growth with EC_50_ values of 63.2 ± 13.1 µM and 86.3 ± 3.3 µM, on MSCs and HS-27a, respectively (Table 3, entries 1 and 2). Interestingly, quinoxaline **5e** did not significantly inhibit HS-27a and MSC cell growth (EC_50_ values > 100 µM) (Table 3, entry 3), suggesting a selective effect of this compound on myeloid leukemia cell growth, sparing normal bone marrow stromal cells of the leukemic niche.

The best candidates were finally evaluated on the human colorectal carcinoma cell line HCT-116, a solid tumor cell line, highly expressing Pim-1 and Pim-2 kinases [12,37], using an MTT assay. Compounds **1**, **5c**, and **5e** exhibited the same level of activity on this cell line with EC_50_ values ranging from 32.9 ± 10.2 µM to 45.3 ± 1.0 µM (Table 3, entries 1, 2 and 3).

It is interesting to notice that, even if the reference drug SGI-1776 demonstrated higher activity on leukemic and colorectal carcinoma cells’ growth than our derivatives, this compound also potently inhibited MSC and HS-27a cells’ growth (Table 3, entry 4), which is consistent with its lack of selectivity on the selected panel of mammalian protein kinases studied (Table 2).

## 3. Materials and Methods

### 3.1. General Remarks

All solvents were anhydrous reagents from commercial sources. Unless otherwise noted, all chemicals and reagents were obtained commercially and used without purification. Microwave heating was carried out with a single-mode Initiator Alstra (Biotage) unit. Melting points (Mp) were determined on a Stuart capillary apparatus and are uncorrected. High-resolution mass spectra (HRMS) were performed in positive mode with an ESI source on a Q-TOF mass spectrometer (Bruker maXis) with an accuracy tolerance of 2 ppm. NMR spectra were recorded at 300 MHz (^1^H) or 75 MHz (^13^C) on a Bruker Avance (300 MHz) spectrometer. The chemical shifts are reported in parts per million (ppm, δ) relative to residual deuterated solvent peaks. The abbreviations s = singlet, d = doublet, t = triplet, q = quadruplet, qt = quintuplet, m = multiplet and bs = broad signal were used throughout. In the ^1^H-NMR spectra of quinoxalines **5a**–**5h**, the carboxylic acid proton was not observed. The presence of the carbonyl peak has been confirmed by ^13^C-NMR and the compound structure by HRMS. The identification of compounds **2b**, **2d**, **2f**, and **2g** was realized by comparison with literature spectroscopic data [30,31]. The reference of the literature-relevant spectroscopic data is given below the characterization of each concerned compound.

### 3.2. Chemistry

*Ethyl 6-fluoro-3-oxo-3,4-dihydroquinoxaline-2-carboxylate (**2a**) and ethyl 7-fluoro-3-oxo-3,4-dihydroquinoxaline-2-carboxylate (**2b**)*, Method A: a mixture of 4-fluoro-1,2-phenylenediamine (366.8 mg, 2.91 mmol), diethyl 2-oxomalonate (506.5 mg, 2.91 mmol) and citric acid (83.8 mg, 0.44 mmol) in ethanol (15 mL) was stirred magnetically at room temperature overnight. Ethanol was then evaporated under reduced pressure, and the resulting residue was purified by silica column chromatography using cyclohexane with ethyl acetate gradient (0–60%) as eluents to give compounds **2a** (190.6 mg, 28%) and **2b** (372.0 mg, 54%) as yellow powders.

*Compound **2a***: Mp 186.8 °C. ^1^H NMR (300 MHz, DMSO-*d6*) δ 12.96 (bs, 1H, NH), 7.91 (dd, 1H, *J* = 9.0, 5.7 Hz), 7.24 (ddd, 1H, *J* = 9.0, 2.7 Hz), 7.07 (dd, 1H, *J* = 9.0, 2.7 Hz), 4.36 (q, 2H, *J* = 7.1 Hz, CH_2_), 1.32 (t, 3H, *J* = 7.1 Hz, CH_3_). ^13^C NMR (75 MHz, DMSO-*d6*) δ 164.0 (d, *J* = 248.9 Hz), 163.9, 152.7, 149.8, 135.0 (d, *J* = 13.0 Hz), 132.4 (d, *J* = 11.0 Hz), 128.3 (d, *J* = 1.5 Hz), 112.7 (d, *J* = 24.2 Hz), 102.2 (d, *J* = 26.7 Hz), 62.3, 14.4. ^19^F NMR (282 MHz, DMSO-*d6*) δ −106.0. HRMS (ESI) *m*/*z*: [M+H]^+^ calcd for C_11_H_10_FN_2_O_3_, 237.0670; found: 237.0668.

*Compound **2b***: Mp 207.6 °C. ^1^H NMR (300 MHz, DMSO-*d6*) δ 12.97 (bs, 1H, NH), 7.73 (dd, 1H, *J* = 9.0, 2.9 Hz), 7.59 (ddd, 1H, *J* = 9.0, 2.9 Hz), 7.38 (dd, 1H, *J =* 9.0, 5.1 Hz), 4.38 (q, 2H, *J* = 7.1 Hz, CH_2_), 1.32 (t, 3H, *J* = 7.1 Hz, CH_3_). ^13^C NMR (75 MHz, DMSO-*d6*) δ 163.9, 158.4 (d, *J* = 239.3 Hz), 152.5, 152.3, 131.4 (d, *J* = 11.6 Hz), 130.1, 120.9 (d, *J* = 24.6 Hz), 117.8 (d, *J* = 9.2 Hz), 114.7 (d, *J* = 22.7 Hz), 62.4, 14.4. ^19^F NMR (282 MHz, DMSO-*d6*) δ −118.4. HRMS (ESI) *m*/*z*: [M+H]^+^ calcd for C_11_H_10_FN_2_O_3_, 237.0670; found: 237.0667.

Literature spectroscopic data reference [30].

*Ethyl 6-chloro-3-oxo-3,4-dihydroquinoxaline-2-carboxylate (**2c**) and ethyl 7-chloro-3-oxo-3,4-dihydroquinoxaline-2-carboxylate (**2d**)*: The title compound was synthesized according to the general method A from 4-chloro-1,2-phenylenediamine (285.2 mg, 2.00 mmol), diethyl 2-oxomalonate (348.3 mg, 2.00 mmol) and citric acid (57.7 mg, 0.30 mmol) in ethanol (10 mL). The reaction mixture was stirred magnetically at room temperature for 2 h. The solvent was then removed under reduced pressure, and the resulting residue was purified by silica column chromatography using cyclohexane with ethyl acetate gradient (0–40%) as eluent to give compounds **2c** (161.7 mg, 32%) and **2d** (274.2 mg, 54%) as yellow powders.

*Compound **2c***: Mp 236.0 °C. ^1^H NMR (300 MHz, DMSO-*d6*) δ 12.94 (bs, 1H, NH), 7.85 (d, 1H, *J* = 8.7 Hz), 7.40 (dd, 1H, *J* = 8.7, 2.2 Hz), 7.34 (d, 1H, *J* = 2.2 Hz), 4.37 (q, 2H, *J* = 7.1 Hz, CH_2_), 1.32 (t, 3H, *J* = 7.1 Hz, CH_3_). ^13^C NMR (75 MHz, DMSO-*d6*) δ 163.8, 152.6, 151.1, 136.8, 134.3, 131.4, 130.0, 124.5, 115.6, 62.3, 14.4. HRMS (ESI) *m*/*z*: [M+H]^+^ calcd for C_11_H_10_ClN_2_O_3_: 253.0374; found: 253.0374.

*Compound **2d***: Mp 213.2 °C. ^1^H NMR (300 MHz, DMSO-*d6*) δ 13.00 (bs, 1H, NH), 7.93 (d, 1H, *J* = 2.3 Hz), 7.69 (dd, 1H, *J* = 8.8, 2.3 Hz), 7.36 (d, 1H, *J* = 8.8 Hz), 4.38 (q, 2H, *J* = 7.1 Hz, CH_2_), 1.32 (t, 3H, *J* = 7.1 Hz, CH_3_). ^13^C NMR (75 MHz, DMSO-*d6*) δ 163.8, 152.6, 152.2, 132.6, 132.2, 131.7, 128.6, 128.0, 118.0, 62.4, 14.4. HRMS (ESI) *m*/*z*: [M+H]^+^ calcd for C_11_H_10_ClN_2_O_3_: 253.0374; found: 253.0373.

Literature spectroscopic data reference [30].

*Ethyl 6-bromo-3-oxo-3,4-dihydroquinoxaline-2-carboxylate (**2e**) and ethyl 7-bromo-3-oxo-3,4-dihydroquinoxaline-2-carboxylate (**2f**)*: The title compounds were synthesized according to the general method A from 4-bromo-1,2-phenylenediamine (748.2 mg, 4.00 mmol), diethyl 2-oxomalonate (696.6 mg, 4.00 mmol) and citric acid (115.3 mg, 0.60 mmol) in ethanol (15 mL). The reaction mixture was stirred magnetically at room temperature for 24 h. The solvent was then removed under reduced pressure, and the resulting residue was purified by silica column chromatography using cyclohexane with ethyl acetate gradient (40–70%) as eluent to give compound **2e** (393.5 mg, 33%) as a beige powder and compound **2f** (304.5 mg, 26%) as an orange powder.

*Compound **2e***: Mp 234.8°C. ^1^H NMR (300 MHz, DMSO-*d6*) δ 12.93 (bs, 1H, NH), 7.77 (d, 1H, *J* = 8.6 Hz), 7.52 (dd, 1H, *J* = 8.6, 2.0 Hz), 7.49 (d, 1H, *J* = 2.0 Hz), 4.36 (q, 2H, *J* = 7.1 Hz, CH_2_), 1.31 (t, 3H, *J* = 7.1 Hz, CH_3_). ^13^C NMR (75 MHz, DMSO-*d6*) δ 163.9, 152.6, 151.3, 134.5, 131.5, 130.2, 127.3, 125.6, 118.6, 62.3, 14.4. HRMS (ESI) *m*/*z*: [M+H]^+^ calcd for C_11_H_10_BrN_2_O_3_: 296.9869; found: 296.9869.

*Compound **2f***: Mp 234.8°C. ^1^H NMR (300 MHz, DMSO-*d6*) δ 13.00 (bs, 1H, NH), 8.06 (d, 1H, *J* = 2.2 Hz), 7.80 (dd, 1H, *J* = 8.8, 2.2 Hz), 7.29 (d, 1H, *J* = 8.8 Hz), 4.37 (q, 2H, *J* = 7.1 Hz, CH_2_), 1.32 (t, 3H, *J* = 7.1 Hz, CH_3_). ^13^C NMR (75 MHz, DMSO-*d6*) δ 163.8, 152.6, 152.1, 135.2, 132.6, 132.1, 131.6, 118.2, 115.6, 62.4, 14.4. HRMS (ESI) *m*/*z*: [M+H]^+^ calcd for C_11_H_10_BrN_2_O_3_: 296.9869; found: 296.9868.

Literature spectroscopic data reference [30].

*Ethyl 3-oxo-6-(trifluoromethyl)-3,4-dihydroquinoxaline-2-carboxylate (**2g**) and ethyl 3-oxo-7-(trifluoromethyl)-3,4-dihydroquinoxaline-2-carboxylate (**2h**)*: The title compounds were synthesized according to the general method A from 4-trifluoromethyl-1,2-phenylenediamine (387.5 mg, 2.20 mmol), diethyl 2-oxomalonate (383.1 mg, 2.20 mmol) and citric acid (63.4 mg, 0.33 mmol) in ethanol (15 mL). The reaction mixture was stirred magnetically at room temperature overnight. The solvent was then removed under reduced pressure, and the resulting residue was purified by silica column chromatography using dichloromethane with ethyl acetate gradient (0–20%) as eluent to give the desired compounds **2g** (325.8 mg, 52%) and **2h** (98.9 mg, 16%) as beige powders.

*Compound **2g***: Mp 204.1 °C. ^1^H NMR (300 MHz, DMSO-*d6*) δ 13.11 (bs, 1H, NH), 8.06 (d, 1H, *J* = 8.4 Hz), 7.67 (dd, 1H, *J* = 8.4, 1.8 Hz), 7.63 (d, 1H, *J* = 1.8 Hz), 4.40 (q, 2H, *J* = 7.2 Hz, CH_2_), 1.33 (t, 3H, *J* = 7.2 Hz, CH_3_). ^13^C NMR (75 MHz, DMSO-*d6*) δ 163.7, 153.6, 152.6, 133.4, 133.0, 131.7 (q, *J* = 32.3 Hz), 131.1, 124.0 (q, *J* = 271.0 Hz), 120.3 (q, *J* = 3.5 Hz), 113.5 (q, *J* = 3.8 Hz), 62.5, 14.4. ^19^F RMN (282 MHz, DMSO-*d6*) δ −61.4. HRMS (ESI) *m*/*z*: [M+H]^+^ calcd for C_12_H_10_F_3_N_2_O_3_: 287.0638; found: 287.0637.

Literature spectroscopic data reference [31].

*Compound **2h***: Mp 161.2 °C. ^1^H NMR (300 MHz, DMSO-*d6*) δ 13.18 (bs, 1H, NH), 8.20 (d, 1H, *J* = 1.5 Hz), 7.96 (dd, 1H, *J* = 8.7, 1.5 Hz), 7.51 (d, 1H, *J* = 8.7 Hz), 4.39 (q, 2H, *J* = 7.2 Hz, CH_2_), 1.33 (t, 3H, *J* = 7.2 Hz, CH_3_). ^13^C NMR (75 MHz, DMSO-*d6*) δ 163.7, 152.9, 152.6, 136.1, 130.4, 128.7 (q, *J* = 3.3 Hz), 127.0 (q, *J* = 4.1 Hz), 124.6 (q, *J* = 32.7 Hz), 124.3 (q, *J* = 270.2 Hz), 117.7, 62.5, 14.4. ^19^F RMN (282 MHz, DMSO-*d6*) δ −60.4. HRMS (ESI) *m*/*z*: [M+H]^+^ calcd for C_12_H_10_F_3_N_2_O_3_: 287.0638; found: 287.0635.

*Ethyl 3-chloro-6-fluoroquinoxaline-2-carboxylate (**3a**)*, Method B: into a dry three-neck round bottom flask was introduced compound **2a** (141.3 mg, 0.60 mmol) in phosphorous oxychloride (1.4 mL) at ice bath temperature. *N,N*-dimethylformamide (140 µL) was then added at 0 °C and the reaction mixture was refluxed for 3 h. After cooling, the resulting mixture was poured over ice, stirred magnetically at room temperature overnight, and extracted with dichloromethane. The combined organic layers were dried over MgSO_4_, filtered, and evaporated under reduced pressure to obtain derivative **3a** (112.9 mg, 74%) as a brown oil. ^1^H NMR (300 MHz, DMSO-*d6*) δ 8.32 (dd, 1H, *J* = 9.2, 5.8 Hz), 8.02–7.90 (m, 2H), 4.49 (q, 2H, *J* = 7.1 Hz, CH_2_), 1.38 (t, 3H, *J* = 7.1 Hz, CH_3_). ^19^F RMN (282 MHz, DMSO-*d6*) δ −103.6.

*Ethyl 3-chloro-7-fluoroquinoxaline-2-carboxylate (**3b**)*: The title compound was synthesized according to the general method B from compound **2b** (236.2mg, 1.00 mmol), phosphorous oxychloride (2.3 mL) and DMF (230 µL). Compound **3b** was obtained (254.6 mg, 100%) as a brown oil. ^1^H NMR (300 MHz, DMSO-*d6*) δ 8.23 (dd, 1H, *J* = 9.2, 5.6 Hz, 1H), 8.09 (dd, 1H, *J* = 9.2, 2.8 Hz), 8.00 (ddd, 1H, *J* = 9.2, 8.4, 2.8 Hz), 4.50 (t, 2H, *J* = 7.1 Hz, CH_2_), 1.39 (t, 3H, *J* = 7.1 Hz, CH_3_). ^19^F RMN (282 MHz, DMSO-*d6*) δ −105.8.

*Ethyl 3,6-dichloroquinoxaline-2-carboxylate (**3c**)*: The title compound was synthesized according to the general method B from compound **2c** (50.0 mg, 0.20 mmol), phosphorous oxychloride (461 µL) and DMF (46 µL). Compound **3c** was obtained (53.7 mg, 100%) as a brown oil. ^1^H NMR (300 MHz, DMSO-*d6*) δ 8.29 (d, 1H, *J* = 2.3 Hz), 8.26 (d, 1H, *J* = 9.0 Hz), 8.03 (dd, 1H, *J* = 9.0, 2.3 Hz), 4.50 (q, 2H, *J* = 7.1 Hz, CH_2_), 1.39 (t, 3H, *J* = 7.1 Hz, CH_3_).

*Ethyl 3,7-dichloroquinoxaline-2-carboxylate (**3d**)*: The title compound was synthesized according to the general method B from compound **2d** (200.0 mg, 0.79 mmol), phosphorous oxychloride (1.9 mL) and DMF (185 µL). Compound **3d** was obtained (202.0 mg, 94%) as a brown oil. ^1^H NMR (300 MHz, DMSO-*d6*) δ 8.37 (d, 1H, *J* = 2.2 Hz), 8.17 (d, 1H, *J* = 9.0 Hz), 8.07 (dd, 1H, *J* = 9.0, 2.2 Hz), 4.51 (q, 1H, *J* = 7.1 Hz, CH_2_), 1.39 (t, 1H, *J* = 7.1 Hz, CH_3_).

*Ethyl 6-bromo-3-chloroquinoxaline-2-carboxylate (**3e**)*: The title compound was synthesized according to the general method B from compound **2e** (200.0 mg, 0.67 mmol), phosphorous oxychloride (1.6 mL) and DMF (157 µL). Compound **3e** was obtained (212.4 mg, 100%) as a brown oil. ^1^H NMR (300 MHz, DMSO-*d6*) δ 8.39 (dd, 1H, *J* = 1.9, 0.8 Hz), 8.16–8.08 (m, 2H), 4.49 (q, 2H, *J* = 7.1 Hz, CH_2_), 1.38 (t, 3H, *J* = 7.1 Hz, CH_3_).

*Ethyl 7-bromo-3-chloroquinoxaline-2-carboxylate (**3f**)*: The title compound was synthesized according to the general method B from compound **2f** (200.0 mg, 0.67 mmol), phosphorous oxychloride (1.6 mL) and DMF (157 µL). Compound **3f** was obtained (212.2 mg, 100%) as a brown oil. ^1^H NMR (300 MHz, DMSO-*d6*) δ 8.52 (dd, 1H¸ *J* = 2.1, 0.5 Hz), 8.18 (dd, 1H¸ *J* = 9.0, 2.1 Hz), 8.08 (dd, 1H, *J* = 9.0, 0.5 Hz), 4.50 (q, 2H, *J* = 7.1 Hz, CH_2_), 1.38 (t, 3H, *J* = 7.1 Hz, CH_3_).

*Ethyl 3-chloro-6-(trifluoromethyl)quinoxaline-2-carboxylate (**3g**)*: The title compound was synthesized according to the general method B from compound **2g** (278.0 mg, 0.97 mmol), phosphorous oxychloride (2.3 mL) and DMF (230 µL). Compound **3g** was obtained (295.8 mg, 100%) as a yellow oil. ^1^H NMR (300 MHz, DMSO-*d6*) δ 8.57 (d, 1H, *J* = 2.0 Hz), 8.44 (d, 1H, *J* = 8.8 Hz), 8.25 (dd, 1H, *J* = 8.8, 2.0 Hz), 4.52 (q, 2H, *J* = 7.1 Hz, CH_2_), 1.40 (t, 3H, *J* = 7.1 Hz, CH_3_). ^19^F RMN (282 MHz, DMSO-*d6*) δ −61.3.

*Ethyl 3-chloro-7-(trifluoromethyl)quinoxaline-2-carboxylate (**3h**)*: The title compound was synthesized according to the general method B from compound **2h** (50.0 mg, 0.18 mmol), phosphorous oxychloride (0.4 mL) and DMF (40 µL). Compound **3h** was obtained (27.0 mg, 51%) as a yellow oil. ^1^H NMR (300 MHz, DMSO-*d6*) δ 8.64 (d, 1H, *J* = 0.8 Hz), 8.33 (d, 1H, *J* = 8.9 Hz), 8.28 (dd, 1H, *J* = 8.9, 0.8 Hz), 4.52 (q, 2H, *J* = 7.1 Hz, CH_2_), 1.39 (t, 3H, *J* = 7.1 Hz, CH_3_). ^19^F RMN (282 MHz, DMSO-*d6*) δ −61.3 (s).

*Ethyl 6-fluoro-3-((3-hydroxyphenyl)amino)quinoxaline-2-carboxylate (**4a**)*, Method C1: a solution of compound **3a** (84.6 mg, 0.33 mmol) and 3-aminophenol (39.8 mg, 0.365 mmol) in absolute ethanol (2 mL) was stirred under microwave irradiation for 3 h at 150 °C. Ethanol was then evaporated under reduced pressure, and the resulting residue was purified by silica column chromatography using cyclohexane with ethyl acetate gradient (0–30%) as eluent to give compound **4a** (88.9 mg, 82%) as an orange powder. Mp 199.8 °C. ^1^H NMR (300 MHz, DMSO-*d6*) δ 10.15 (bs, 1H), 9.49 (bs, 1H), 8.06 (ddd, 1H, *J* = 9.1, 6.1, 0.5 Hz), 7.55 (t, 1H, *J* = 2.0 Hz), 7.53–7.42 (m, 2H), 7.24–7.14 (m, 2H), 6.53 (ddd, 1H, *J* = 7.7, 2.0, 1.3 Hz), 4.48 (q, 2H, *J* = 7.1 Hz, CH_2_), 1.41 (t, 3H, *J* = 7.1 Hz, CH_3_). ^13^C NMR (75 MHz, DMSO-*d6*) δ 165.9, 164.9 (d, *J* = 247.5 Hz), 158.3, 149.2, 143.7 (d, *J* = 14.6 Hz), 140.3, 133.3, 132.7 (d, *J* = 11.0 Hz), 132.2, 130.1, 116.5 (d, *J* = 25.6 Hz), 111.4, 110.9, 110.4 (d, *J* = 21.8 Hz), 107.6, 60.2, 14.5. ^19^F RMN (282 MHz, DMSO-*d6*) δ −105.3. HRMS (ESI) *m*/*z*: [M+H]^+^ calcd for C_17_H_15_FN_3_O_3_: 328.1092; found: 328.1094.

*Ethyl 7-fluoro-3-((3-hydroxyphenyl)amino)quinoxaline-2-carboxylate (**4b**)*: The title compound was synthesized according to the general method C1 from compound **3b** (254.6 mg, 1.00 mmol) and 3-aminophenol (120.0 mg, 1.10 mmol) in absolute ethanol (5 mL). Compound **4b** was obtained (138.6 mg, 42%) as a red powder. Mp 223.4 °C. ^1^H NMR (300 MHz, DMSO-*d6*) δ 10.01 (bs, 1H), 9.47 (bs, 1H), 7.85–7.70 (m, 3H), 7.55 (t, 1H, *J* = 1.8 Hz), 7.22–7.13 (m, 2H), 6.51 (ddd, 1H, *J* = 7.3, 1.8 Hz), 4.48 (q, 2H, *J* = 7.1 Hz, CH_2_), 1.41 (t, 3H, *J* = 7.1 Hz, CH_3_). ^13^C NMR (75 MHz, DMSO-*d6*) δ 165.8, 160.1 (d, *J* = 243.1 Hz), 158.3, 148.5, 140.6, 139.6, 136.0 (d, *J* = 12.8 Hz), 133.6, 130.0, 128.7 (d, *J* = 9.6 Hz), 123.1 (d, *J* = 26.7 Hz), 113.5 (d, *J* = 21.4 Hz), 111.2, 110.6, 107.3, 62.9, 14.5. ^19^F RMN (282 MHz, DMSO-*d6*) δ -114.4. HRMS (ESI) *m*/*z*: [M+H]^+^ calcd for C_17_H_15_FN_3_O_3_: 328.1092; found: 328.1092.

*Ethyl 6-chloro-3-((3-hydroxyphenyl)amino)quinoxaline-2-carboxylate (**4c**)*: The title compound was synthesized according to the general method C1 from compound **3c** (203.1 mg, 0.75 mmol) and 3-aminophenol (245.2 mg, 2.25 mmol) in absolute ethanol (5 mL). After purification by silica column chromatography using cyclohexane with ethyl acetate gradient (0–40%) as eluent, compound **4c** was obtained (253.9 mg, 99%) as an orange powder. Mp 226.8 °C. ^1^H NMR (300 MHz, DMSO-*d6*) δ 10.14 (bs, 1H), 9.48 (bs, 1H), 8.01 (d, 1H, *J* = 8.9 Hz), 7.82 (dd, 1H, *J* = 2.3, 1.0), 7.63 (m, 1H), 7.57 (dd, 1H, *J* = 8.9, 2.2 Hz), 7.22–7.14 (m, 2H), 6.56–6.50 (m, 1H), 4.48 (q, 2H, *J* = 7.1 Hz, CH_2_), 1.40 (t, 3H, *J* = 7.1 Hz, CH_3_). ^13^C NMR (75 MHz, DMSO-*d6*) δ 165.8, 158.3, 149.2, 142.9, 140.3, 137.8, 134.6, 133.3, 131.8, 130.1, 127.0, 125.3, 111.3, 110.9, 107.5, 62.9, 14.5. HRMS (ESI) *m*/*z*: [M+H]^+^ calcd for C_17_H_15_ClN_3_O_3_: 344.0796; found: 344.0794.

*Ethyl 7-chloro-3-((3-hydroxyphenyl)amino)quinoxaline-2-carboxylate (**4d**)*: The title compound was synthesized according to the general method C1 from compound **3d** (167 mg, 0.62 mmol) and 3-aminophenol (74 mg, 0.68 mmol) in absolute ethanol (5 mL). After purification by silica column chromatography using dichloromethane with ethyl acetate gradient (0–10%) as eluent, compound **4d** was obtained (192 mg, 91%) as a red powder. Mp 216.9 °C. ^1^H NMR (300 MHz, DMSO-*d6*) δ 10.07 (bs, 1H), 9.49 (bs, 1H), 8.06 (d, 1H, *J* = 2.2 Hz), 7.82 (dd, 1H, *J* = 8.8, 2.2 Hz), 7.77 (d, 1H, *J* = 8.8 Hz), 7.56 (t, 1H, *J* = 2.1 Hz), 7.22–7.14 (m, 2H), 6.52 (ddd, 1H, *J* = 7.0, 2.1 Hz), 4.48 (q, 2H, *J* = 7.1 Hz, CH_2_), 1.40 (t, 3H, *J* = 7.1 Hz, CH_3_). ^13^C NMR (75 MHz, DMSO-*d6*) δ 165.7, 158.3, 148.8, 141.1, 140.4, 136.1, 133.9, 133.6, 130.4, 130.0, 128.5, 128.3, 111.3, 110.8, 107.5, 62.9, 14.5. HRMS (ESI) *m*/*z*: [M+H]^+^ calcd for C_17_H_15_ClN_3_O_3_: 344.0796; found: 344.0799.

*Ethyl 6-bromo-3-((3-hydroxyphenyl)amino)quinoxaline-2-carboxylate (**4e**)*: The title compound was synthesized according to the general method C1 from compound **3e** (203.2 mg, 0.64 mmol) and 3-aminophenol (210.8 mg, 1.93 mmol) in absolute ethanol (5 mL). After cooling, the reaction mixture was filtered, giving compound **4e** (72.4 mg, 29%) as a red powder. Mp 230.6 °C. ^1^H NMR (300 MHz, DMSO-*d6*) δ 10.14 (bs, 1H), 9.48 (bs, 1H), 7.98 (d, 1H, *J* = 2.1 Hz), 7.91 (d, 1H, *J* = 8.8 Hz), 7.70–7.64 (m, 2H), 7.21–7.13 (m, 2H), 6.55–6.49 (m, 1H), 4.47 (q, 2H, *J* = 7.1 Hz, CH_2_), 1.40 (t, 2H, *J* = 7.1 Hz, CH_3_). ^13^C NMR (75 MHz, DMSO-*d6*) δ 165.8, 158.3, 149.1, 143.1, 140.3, 134.8, 133.3, 131.8, 130.1, 129.6, 128.6, 126.7, 111.3, 110.8, 107.5, 62.9, 14.5. HRMS (ESI) *m*/*z*: [M+H]^+^ calcd for C_17_H_15_BrN_3_O_3_: 388.0291; found: 388.0291.

*Ethyl 7-bromo-3-((3-hydroxyphenyl)amino)quinoxaline-2-carboxylate (**4f**):* The title compound was synthesized according to the general method C1 from compound **3f** (210.9 mg, 0.67 mmol) and 3-aminophenol (218.7 mg, 2.00 mmol) in absolute ethanol (5 mL). After purification by silica column chromatography using cyclohexane with ethyl acetate gradient (0–50%) as eluent, compound **4f** was obtained (88.4 mg, 34%) as a red powder. Mp 244.1 °C. ^1^H NMR (300 MHz, DMSO-*d6*) δ 10.08 (bs, 1H), 9.50 (bs, 1H), 8.20 (d, 1H, *J* = 2.2 Hz), 7.91 (dd, 1H, *J* = 8.9, 2.2 Hz), 7.70 (d, 1H, *J* = 8.9 Hz), 7.58–7.56 (m, 1H), 7.22–7.14 (m, 2H), 6.52 (ddd, 1H, *J* = 7.1, 2.1 Hz), 4.48 (q, 2H, *J* = 7.1 Hz, CH_2_), 1.40 (t, 3H, *J* = 7.1 Hz, CH_3_). ^13^C NMR (75 MHz, DMSO-*d6*) δ 165.7, 158.3, 148.9, 141.4, 140.4, 136.6, 136.1, 133.8, 131.7, 130.0, 128.5, 118.5, 111.3, 110.8, 107.5, 62.9, 14.5. HRMS (ESI) *m*/*z*: [M+H]^+^ calcd for C_17_H_15_BrN_3_O_3_: 388.0291; found: 388.0290.

*Ethyl 3-((3-hydroxyphenyl)amino)-6-(trifluoromethyl)quinoxaline-2-carboxylate (**4g**)*, Method C2: a solution of compound **3g** (256.9 mg, 0.84 mmol), 3-aminophenol (276.0 mg, 2.53 mmol) and *p*-TSA, as a catalyst, in absolute ethanol (1.5 mL) was stirred under microwave irradiation for 8 h at 100 °C. Ethanol was then evaporated under reduced pressure, and the resulting residue was purified by silica column chromatography using cyclohexane with ethyl acetate gradient (0–30%) as eluent to give the desired compound **4g** (222.7 mg, 70%) as a red powder. Mp 184.7 °C. ^1^H NMR (300 MHz, DMSO-*d6*) δ 10.16 (bs, 1H), 9.52 (bs, 1H), 8.20 (d, 1H, *J* = 8.6 Hz), 8.10 (d, 1H, *J* = 1.8 Hz), 7.79 (dd, 1H, *J* = 8.6, 1.8 Hz), 7.67 (m, 1H), 7.24–7.16 (m, 2H), 6.54 (ddd, 1H, *J* = 6.9, 2.4 Hz), 4.50 (q, 2H, *J* = 7.2 Hz, CH_2_), 1.42 (t, 3H, *J* = 7.2 Hz, CH_3_). ^13^C NMR (75 MHz, DMSO-*d6*) δ 165.5, 158.3, 149.2, 141.7, 140.2, 137.2, 135.6, 132.7 (q, *J* = 31.9 Hz), 131.6, 130.1, 124.2 (q, *J* = 271.1 Hz), 124.1 (q, *J* = 3.5 Hz), 121.6 (q, *J* = 4.0 Hz), 111.4, 111.0, 107.6, 63.1, 14.4. ^19^F RMN (282 MHz, DMSO-d6) δ −61.4. HRMS (ESI) *m*/*z*: [M+H]^+^ calcd for C_18_H_15_F_3_N_3_O_3_: 378.1060; found: 378.1059.

*Ethyl 3-((3-hydroxyphenyl)amino)-7-(trifluoromethyl)quinoxaline-2-carboxylate (**4h**)*: The title compound was synthesized according to the general method C1 from compound **3h** (86.2 mg, 0.28 mmol) and 3-aminophenol (33.9 mg, 0.31 mmol) in absolute ethanol (2 mL). After purification by silica column chromatography using cyclohexane with ethyl acetate gradient (0–20%) as eluent, compound **4h** was obtained (38.8 mg, 36%) as a yellow powder. Mp 178.5 °C. ^1^H NMR (300 MHz, DMSO-*d6*) δ 10.22 (bs, 1H), 9.56 (bs, 1H), 8.34 (d, 1H, *J* = 1.8 Hz), 8.05 (dd, 1H, *J* = 8.7, 1.8 Hz), 7.93 (d, 1H, *J* = 8.7 Hz), 7.60 (m, 1H), 7.23–7.17 (m, 2H), 6.56 (ddd, 1H, *J* = 7.2, 1.8 Hz), 4,50 (q, 2H, *J* = 7.1 Hz, CH_2_), 1.42 (t, 3H, *J* = 7.1 Hz, CH_3_). ^13^C NMR (75 MHz, DMSO-*d6*) δ 165.5, 158.3, 149.7, 144.2, 140.1, 134.8, 134.7, 130.1, 128.4 (q, *J* = 3.1 Hz), 128.1, 127.7 (q, *J* = 4.4 Hz), 126.3 (q, *J* = 32.3 Hz), 124.4 (q, *J* = 270.0 Hz), 111.6, 111.2, 107.8, 63.0, 14.4. ^19^F RMN (282 MHz, DMSO-*d6*) δ −60.6 (t, *J* = 2.9 Hz). HRMS (ESI) *m*/*z*: [M+H]^+^ calcd for C_18_H_15_F_3_N_3_O_3_: 378.1060; found: 378.1059.

*6-Fluoro-3-((3-hydroxyphenyl)amino)quinoxaline-2-carboxylic acid (**5a**)*, Method D: to ester **4a** (43.2 mg, 0.13 mmol) in aqueous methanol (80%, 15 mL), potassium carbonate (54.7 mg, 0.40 mmol) was added and the reaction mixture was refluxed for 4 h. After cooling, the solvent was removed under reduced pressure. Then, the residue was acidified with a 15% hydrochloric acid aqueous solution, and extracted with dichloromethane. The combined organic layers were washed with brine, dried over MgSO_4_, filtered, and evaporated under reduced pressure to yield the acid **5a** (39.5 mg, 100%) as a dark red powder. Mp 200.4 °C. ^1^H NMR (300 MHz, DMSO-*d6*) δ 10.59 (bs, 1H), 9.47 (bs, 1H), 8.04 (m, 1H), 7.56 (t, 1H *J* = 2.0 Hz), 7.52–7.40 (m, 2H), 7.24 (ddd, 1H, *J* = 7.9, 2.0, 1.0 Hz), 7.17 (t, 1H, *J* = 7.9 Hz), 6.52 (ddd, 1H, *J* = 7.9, 2.0, 1.0 Hz). ^13^C NMR (75 MHz, DMSO-*d6*) δ 168.0, 164.8 (d, *J* = 249.7 Hz), 158.3, 149.7, 144.0, 143.8, 140.4, 133.2, 132.5 (d, *J* = 11.6 Hz), 130.1, 116.2 (d, *J* = 25.9 Hz), 111.2, 110.8, 110.3 (d, *J* = 21.8 Hz), 107.4. ^19^F RMN (282 MHz, DMSO-*d6*) δ −105.6. HRMS (ESI) *m*/*z*: [M+H]^+^ calcd for C_15_H_11_FN_3_O_3_: 300.0779; found: 300.0779.

*7-Fluoro-3-((3-hydroxyphenyl)amino)quinoxaline-2-carboxylic acid (**5b**)*: The title compound was synthesized according to the general method D from compound **4b** (107.3 mg, 0.33 mmol) and potassium carbonate (135.9 mg, 0.98 mmol) in aqueous methanol (80%, 15 mL). Compound **5b** was obtained (97.1 mg, 99%) as a red powder. Mp 180.1 °C. ^1^H NMR (300 MHz, DMSO-*d6*) δ 10.44 (bs, 1H), 9.47 (bs, 1H), 7.85–7.69 (m, 3H), 7.56 (t, 1H, *J* = 2.0 Hz), 7.22 (ddd, 1H, *J* = 7.7, 2.0, 1.2 Hz), 7.16 (t, 1H, *J* = 7.7 Hz), 6.50 (ddd, 1H, *J* = 7.7, 2.0, 1.2 Hz). ^13^C NMR (75 MHz, DMSO-*d6*) δ 167.9, 160.0 (d, *J* = 243.1 Hz), 158.3, 149.0, 140.7, 139.7, 136.0 (d, *J* = 12.2 Hz), 133.9, 130.0, 128.6 (d, *J* = 9.5 Hz), 122.9 (d, *J* = 25.7 Hz), 113.4 (d, *J* = 21.2 Hz), 111.0, 110.5, 107.2. ^19^F RMN (282 MHz, DMSO-*d6*) δ −114.7. HRMS (ESI) *m*/*z*: [M+H]^+^ calcd for C_15_H_11_FN_3_O_3_: 300.0779; found: 300.0779.

*6-Chloro-3-((3-hydroxyphenyl)amino)quinoxaline-2-carboxylic acid (**5c**)*: The title compound was synthesized according to the general method D from compound **4c** (29.7 mg, 0.09 mmol) and potassium carbonate (35.7 mg, 0.26 mmol) in aqueous methanol (80%, 5 mL). Compound **5c** was obtained (27.2 mg, 100%) as a dark red powder. Mp 194.6 °C. ^1^H NMR (300 MHz, DMSO-*d6*) δ 10.60 (bs, 1H), 9.48 (bs, 1H), 7.98 (d, 1H, *J* = 8.8 Hz), 7.82 (d, 1H, *J* = 2.3 Hz), 7.64 (t, 1H, *J* = 1.9 Hz), 7.57 (dd, 1H, *J* = 8.8, 2.3 Hz), 7.23–7.13 (m, 2H), 6.51 (ddd, 1H, *J* = 7.2, 1.9 Hz). ^13^C NMR (75 MHz, DMSO-*d6*) δ 167.9, 158.3, 149.6, 143.1, 140.4, 137.6, 134.6, 133.5, 131.7, 130.1, 126.9, 125.3, 111.2, 110.7, 107.4. HRMS (ESI) *m*/*z*: [M+H]^+^ calcd for C_15_H_11_ClN_3_O_3_: 316.0483; found: 316.0484.

*7-Chloro-3-((3-hydroxyphenyl)amino)quinoxaline-2-carboxylic acid (**5d**)*: The title compound was synthesized according to the general method D from compound **4d** (128 mg, 0.37 mmol) and potassium carbonate (154 mg, 1.12 mmol) in aqueous methanol (80%, 15 mL). Compound **5d** was obtained (110 mg, 94%) as a dark red powder. Mp 173–174 °C. ^1^H NMR (300 MHz, DMSO-*d6*) δ 10.48 (bs, 1H), 9.49 (bs, 1H), 8.03 (d, 1H, *J* = 2.2 Hz), 7.84–7.76 (m, 2H), 7.57 (t, 1H, *J* = 1.9 Hz), 7.23–7.14 (m, 2H), 6.53–6.49 (m, 1H). ^13^C NMR (75 MHz, DMSO-*d6*) δ 167.3, 157.8, 148.8, 140.8, 140.0, 135.7, 135.5, 133.0, 129.7, 129.6, 128.0, 127.8, 110.7, 110.2, 106.9. HRMS (ESI) *m*/*z*: [M+H]^+^ calcd for C_15_H_11_ClN_3_O_3_: 316.04855; found: 316.04778.

*6-Bromo-3-((3-hydroxyphenyl)amino)quinoxaline-2-carboxylic acid (**5e**)*: The title compound was synthesized according to the general method D from compound **4e** (39.0 mg, 0.10 mmol) and potassium carbonate (41.5 mg, 0.30 mmol) in aqueous methanol (80%, 5 mL). Compound **5e** was obtained (36.0 mg, 100%) as a red powder. Mp 199.0 °C. ^1^H NMR (300 MHz, DMSO-*d6*) δ 10.54 (bs, 1H), 9.47 (bs, 1H), 7.98 (d, 1H, *J* = 2.2 Hz), 7.90 (d, 1H, *J* = 8.8 Hz), 7.70–7.64 (m, 2H), 7.22–7.13 (m, 2H), 6.51 (ddd, 1H, *J* = 6.6, 2.4 Hz). ^13^C NMR (75 MHz, DMSO-*d6*) δ 167.9, 158.3, 149.5, 143.3, 140.4, 134.7, 133.4, 131.7, 130.1, 129.5, 128.6, 126.6, 111.2, 110.7, 107.4. HRMS (ESI) *m*/*z*: [M+H]^+^ calcd for C_15_H_11_BrN_3_O_3_: 359.9978; found: 359.9977.

*7-Bromo-3-((3-hydroxyphenyl)amino)quinoxaline-2-carboxylic acid (**5f**)*: The title compound was synthesized according to the general method D from compound **4f** (40.7 mg, 0.11 mmol) and potassium carbonate (43.5 mg, 0.32 mmol) in aqueous methanol (80%, 5 mL). Compound **5f** was obtained (37.8 mg, 100%) as an orange powder. Mp 193.1 °C. ^1^H NMR (300 MHz, DMSO-*d6*) δ 10.48 (bs, 1H), 9.53 (bs, 1H), 8.16 (d, 1H, *J* = 2.3 Hz), 7.91 (dd, 1H, *J* = 8.9, 2.3 Hz), 7.71 (d, 1H, *J* = 8.9 Hz), 7.58 (t, 1H, *J* = 1.9 Hz), 7.23–7.13 (m, 2H), 6.52 (dd, 1H, *J* = 7.5, 1.9 Hz). ^13^C NMR (75 MHz, DMSO-*d6*) δ 167.8, 158.3, 149.3, 141.5, 140.4, 136.6, 136.0, 133.9, 131.7, 130.1, 128.5, 118.3, 111.1, 110.7, 107.4. HRMS (ESI) *m*/*z*: [M+H]^+^ calcd for C_15_H_11_BrN_3_O_3_: 359.9978; found: 359.9979.

*3-((3-Hydroxyphenyl)amino)-6-(trifluoromethyl)quinoxaline-2-carboxylic acid (**5g**)*: The title compound was synthesized according to the general method D from ester **4g** (30.8 mg, 0.08 mmol) and potassium carbonate (33.6 mg, 0.24 mmol) in aqueous methanol (80%, 15 mL). After cooling, the solvent was removed under reduced pressure. Then, the residue was acidified with a 15% hydrochloric acid aqueous solution, and extracted with diethyl ether. The combined organic layers were washed with brine, dried over MgSO_4_, filtered and evaporated under reduced pressure to yield the acid **5g** (28.6 mg, 100%) as a dark red powder. Mp 196.2 °C. ^1^H NMR (300 MHz, DMSO-*d6*) δ 10.56 (bs, 1H), 9.51 (bs, 1H), 8.17 (d, 1H, *J* = 8.4 Hz), 8.09 (d, 1H, *J* = 1.8 Hz), 7.79 (dd, 1H, *J* = 8.4, 1.8 Hz), 7.68 (m, 1H), 7.24–7.15 (m, 2H), 6.54 (ddd, 1H, *J* = 7.2, 1.8 Hz). ^13^C NMR (75 MHz, DMSO-*d6*) δ 167.7, 158.3, 149.7, 141.8, 140.3, 137.2, 135.7, 132.4 (q, *J* = 37.5 Hz), 131.6, 130.1, 124.3 (q, *J* = 270.0 Hz), 124.1 (q, *J* = 3.0 Hz), 121.4 (q, *J* = 2.3 Hz), 111.3, 110.9, 107.4. ^19^F RMN (282 MHz, DMSO-*d6*) δ −61.4. HRMS (ESI) *m*/*z*: [M+H]^+^ calcd for C_16_H_11_F_3_N_3_O_3_: 350.0747; found: 350.0749.

*3-((3-Hydroxyphenyl)amino)-7-(trifluoromethyl)quinoxaline-2-carboxylic acid (5h)*: The title compound was synthesized according to the general method D from ester **4h** (30.8 mg, 0.08 mmol) and potassium carbonate (33.6 mg, 0.25 mmol) in aqueous methanol (80%, 15 mL). After cooling, the solvent was removed under reduced pressure. Then, the residue was acidified with a 15% hydrochloric acid aqueous solution, and extracted with diethyl ether. The combined organic layers were washed with brine, dried over MgSO_4_, filtered and evaporated under reduced pressure to yield the acid **5h** (33.2 mg, 100%) as a red ocher powder. Mp 178.7 °C. ^1^H NMR (300 MHz, DMSO-*d6*) δ 10.90 (bs, 1H), 9.52 (bs, 1H), 8.29 (s, 1H), 8.03 (dd, 1H, *J* = 8.8, 1.7 Hz), 7.92 (d, 1H, *J* = 8.8 Hz), 7.61 (t, 1H, *J* = 2.0 Hz), 7.26–7.16 (m, 2H), 6.54 (ddd, 1H, *J* = 7.7, 1.3 Hz). ^13^C NMR (75 MHz, DMSO-*d6*) δ 167.4, 158.3, 150.4, 144.5, 140.3, 136.0, 134.6, 130.1, 128.0 (2 × C), 127.4, 125.9 (q, *J* = 30 Hz), 124.7 (q, *J* = 270 Hz), 111.3, 111.0, 107.5. ^19^F RMN (282 MHz, DMSO-*d6*) δ—60.5. HRMS (ESI) *m*/*z*: [M+H]^+^ calcd for C_16_H_11_F_3_N_3_O_3_: 350.0747; found: 350.0747.

### 3.3. Docking Studies

Molecular modeling studies were performed using SYBYL-X 1.3 software [38] running on a Dell precision T3400 workstation. The three-dimensional structure of compounds **5c** and **5f** (under their carboxylate forms to imitate physiological conditions) were built from a standard fragments library and optimized using the Tripos force field [39] including the electrostatic term calculated from Gasteiger and Hückel atomic charges. Powell’s method available in Maximin2 procedure was used for energy minimization until the gradient value was smaller than 0.001 kcal/(mol*Å). The crystal structure of Pim-1 in complex with 5′-adenylyl-β,γ-imidodiphosphate (AMP-PNP) at 1.6 Å resolution (PDB ID 3A99) [40] was used as template for docking. Water molecules were removed from the coordinates set since no information about conserved water molecules is known for this chemical series in Pim-1. Flexible docking of compounds **5c** and **5f** into the ATP-binding site was performed using GOLD software [41]. The most stable docking models were selected according to the best scored conformation predicted by the Chemscore scoring function implemented in GOLD. Finally, the complexes were energy-minimized using Powell’s method available in Maximin2 procedure with the Tripos force field and a dielectric constant of 4.0, until the gradient value reached 0.1 kcal/mol.Å. Biovia Discovery Studio Visualizer [42] was used for graphical display.

### 3.4. Biology

#### 3.4.1. Mammalian Protein Kinase Assays

Kinase enzymatic activities were assayed with 10 µM ATP in 384-well plates using the luminescent ADP-Glo^TM^ assay (Promega, Madison, WI, USA) according to the recommendations of the manufacturer (see [32] for details on this method). The transmitted signal was measured using the Envision (PerkinElmer, Waltham, MA, USA) microplate luminometer and expressed in Relative Light Unit (RLU). In order to determine the half maximal inhibitory concentration (IC_50_), the assays were performed in duplicate in the absence or presence of increasing doses of the tested compounds. GraphPad Prism6 software (GraphPad Software, San Diego, CA, USA) was used to fit dose–response curves and to determine the IC_50_ values. Kinase activities are expressed in % of maximal activity, i.e., measured in the absence of inhibitor. Peptide substrates were obtained from Proteogenix (Schiltigheim, France).

The following kinases were analyzed during this study: *Hs*Pim-1 and *Hs*Pim-2 (human proto-oncogene, recombinant, expressed in bacteria) were assayed with 0.20 µg/µL of consensus peptide substrate: ARKRRRHPSGPPTA; *Rn*DYRK1A-kd (*Rattus norvegicus*, amino acids 1 to 499 including the kinase domain, recombinant, expressed in bacteria, DNA vector kindly provided by Dr. W. Becker, Aachen, Germany) was assayed with 0.033 μg/µL of the following peptide: KKISGRLSPIMTEQ as substrate; *Hs*CDK2/CyclinA (human cyclin-dependent kinase-2, kindly provided by Dr. A. Echalier-Glazer, Leicester, UK) was assayed with 0.8 µg/µL of histone H1 as substrate; *Hs*CDK9/CyclinT (human, recombinant, expressed by baculovirus in Sf9 insect cells) was assayed with 0.20 µg/µL of the following peptide: YSPTSPSYSPTSPSYSPTSPSKKKK, as substrate; *Hs*Haspin-kd (human, kinase domain, amino acids 470 to 798, recombinant, expressed in bacteria) was assayed with 0.007 µg/µL of Histone H3 (1–21) peptide (ARTKQTARKSTGGKAPRKQLA) as substrate; *Mm*CLK1 (from *Mus musculus*, recombinant, expressed in bacteria) was assayed with 0.027 μg/µL of the following peptide: GRSRSRSRSRSR as substrate; *Hs*CK1ε (human casein kinase 1ε, recombinant, expressed by baculovirus in Sf9 insect cells) was assayed with 0.02 µg/µL of the following peptide: RRKHAAIGSpAYSITA (“Sp” stands for phosphorylated serine) as CK1-specific substrate; *Hs*GSK-3β (human glycogen synthase kinase-3) was assayed with 0.010 µg/µL of GS-1 peptide, a GSK-3-selective substrate (YRRAAVPPSPSLSRHSSPHQSpEDEEE). For kinases expressed in bacteria, *Escherichia coli* BL21 DE3 pLysS strain (Invitrogen, ThermoFisher Scientific, Waltham, MA, USA) was transformed with pGEX-2T-1 (Sigma-Aldrich, St. Louis, MO, USA) containing the coding region of the corresponding kinase. For kinases expressed in Sf9 insect cells, the coding region of the corresponding kinase was cloned in pFastBac^TM^ vector. Purification of the recombinant kinases were performed following the protocol “Bac-to-Bac^®^ Baculovirus Expression System” provided by the manufacturer (Invitrogen, ThermoFisher Scientific, Waltham, MA, USA).

To validate the kinase assay, model inhibitors were used for each tested enzyme: Staurosporine from *Streptomyces* sp. (#S5921, purity ≥ 95%, Sigma-Aldrich) for *Hs*CK1ε; Indirubin-3′-oxime (#I0404, purity ≥ 98%, Sigma-Aldrich) for *Hs*GSK-3β, *Hs*Pim-1 and 2, human cyclin-dependent kinases, *Rn*DYRK1A and *Mm*CLK1; CHR-6494 (#SML0648, purity ≥ 98%, Sigma-Aldrich) for Haspin.

#### 3.4.2. Cell Cultures and Reagents

MV4-11 cell lines were obtained from the *Deutshe Sammlung von Mikroorganismens und Zellkulturen* (DSMZ). HS-27a cell lines were obtained from the American Type Culture Collection (ATCC). All cell lines were cultured in Roswell Park Memorial Institute medium (RPMI), with 10% fetal bovine serum, 1% glutamine, and 1% penicillin/streptomycin at 37 °C and 5% CO_2_. Bone marrow (BM) MSCs from brain-dead donors were isolated and cultured as described [43]. Informed consent was obtained before BM samples were taken. The HCT-116 cell line (colorectal carcinoma) was obtained from ATCC and cultured in McCoy’s medium with 10% fetal bovine serum without antibiotics at 37 °C and 5% CO_2_.

#### 3.4.3. In Vitro Cell-Based Assays

Cell viability was studied using an MTT cell proliferation assay. To determine the concentration effect of the molecules, 0.2 × 10^5^ MV4-11 leukemic cells or 0.1 × 10^5^ HS-27a cells were incubated in 100 μL of RPMI red phenol-free medium (ThermoFisher Scientific), 0.035 × 10^5^ MSCs were incubated in 100 µL of alphaMEM medium (Gibco) and 0.05 × 10^5^ HCT-116 cells were incubated in 100 µL of McCoy’s medium, in 96-well plates for 48 h and then treated with quinoxalines (stock solution at 50 mM in DMSO) or SGI-1776 (SAB Signalway antibody, stock solution at 50 mM in DMSO), as reference, with concentrations ranging from 100 nM to 100 μM for 48 h.

Cells were incubated with 10 μL of MTT working solution (5 g/L of methylthiazolyldiphenyl-tetrazolium bromide from Sigma Aldrich, Lyon, France) during 4 h. Cells were then lysed overnight at 37 °C with 100 μL of 10% sodium dodecyl sulfate (SDS) and 0.003% HCl. Optical density (OD) at 570 nm was measured using a spectrophotometer CLARIOstar^®^ (BMG Labtech, Offenburg, Germany) or Mithras Multimode Microplate Reader LB940 (Berthold, Versailles, France). Living cells were also counted with the trypan blue dye exclusion method. When a dose-dependent activity was observed, EC_50_ values were calculated using Graphpad PRISM 7 software (n = 3 in triplicate). Data were collected from at least three independent experiments and the values reported are means ± standard errors of the mean (SEM).

## 4. Conclusions

In summary, using a structure-based design approach, we have developed a new promising quinoxaline-2-carboxylic acid series of dual Pim-1/2 inhibitors. Starting from the lead compound **1**, moderately active on Pim-2, we significantly improved the inhibition profile on Pim-2 isoform by adding halogenated substituents in position 6. Docking studies demonstrated that this 6-halogenated group was oriented towards the unique hydrophobic pocket of the Pim kinases hinge environment, contributing to increase van der Waals interactions in this area. Two lead compounds, **5c** (6-Cl) and **5e** (6-Br), were then identified, exhibiting submicromolar potency on both Pim-1 and Pim-2 isoforms, with an interesting selectivity profile against the panel of mammalian kinases studied. In vitro cell-based assays on human hematologic (AML) and solid tumor (colorectal carcinoma) cell lines overexpressing Pim-1/2 kinases were then realized, showing growth inhibitory activities at micromolar concentrations. These encouraging results make them promising new leads for further pharmacomodulation studies.

## Figures and Tables

**Table 1:** Enzymatic assays on *Hs*Pim-1 and *Hs*Pim-2 (ADP-Glo method at 10 μM ATP).

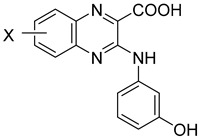				
Entry	Compound	X	*Hs*Pim-1 IC_50_ (µM) ^1^	*Hs*Pim-2 IC_50_ (µM) ^1^
1	**1**	H	0.074	2.10
2	**5a**	6-F	0.17	0.76
3	**5b**	7-F	1.24	4.20
4	**5c**	6-Cl	0.13	0.17
5	**5d**	7-Cl	2.10	2.40
6	**5e**	6-Br	0.16	0.58
7	**5f**	7-Br	0.18	2.20
8	**5g**	6-CF_3_	0.20	1.80
9	**5h**	7-CF_3_	3.85	6.40
10	**SGI-1776**		0.05	0.10

^1^ IC_50_ on Pim-1/2 kinase activity were calculated from dose−response curves. Each inhibitor concentration was tested in duplicate. Inhibition curves for **5c** and **5e** are reported in supplementary data. *Hs*: *Homo sapiens*. Values are a mean of n ≥ 3 independent experiments. *Hs*: *Homo sapiens*.

**Table 2:** Kinase selectivity profile of the most active quinoxalines (ADP-Glo method at 10 μM ATP).

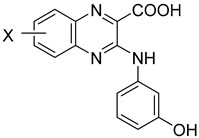											
Entry	Compound	X	Kinase Enzymatic IC_50_ (µM) ^1^								
			Pim-1	Pim-2	DYRK1A	CDK5/p25	CDK9/CyclinT	Haspin	CLK1	CK1ε	GSK3β
1	**1**	H	0.07	2.10	0.27	>10	>10	>10	>10	>10	>10
2	**5c**	6-Cl	0.13	0.17	2.58	>10	>10	>10	>10	>10	2.80
3	**5e**	6-Br	0.16	0.58	1.98	>10	>10	>10	>10	>10	3.22
4	**SGI-1776**		0.05	0.10	3.80	9.53	1.08	0.05	0.43	6.54	>10

^1^ IC_50_ on disease-related kinase activity were calculated from dose−response curves. Each inhibitor concentration was tested in duplicate. Inhibition curves for **5c** and **5e** are reported in supplementary data. All protein kinases used here are human with the exception of DYRK1A (*Rattus norvegicus*) and CLK1 (*Mus musculus*). DYRK1A: dual specificity tyrosine phosphorylation regulated kinase 1A, CDK: cyclin-dependent kinase, Haspin: haploid germ cell-specific nuclear protein kinase, CLK1: CDC2-like kinase 1, CK1: casein kinase 1, GSK3: glycogen synthase kinase 3.

**Table 3:** Cell-based assays of the most active quinoxalines.

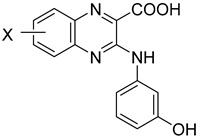						
Entry	Compound	X	EC_50_ (µM) ^1^			
			MV4-11 ^2^	HCT-116 ^2^	HS-27a ^2^	MSC ^2^
1	**1**	H	61.2 ± 3.9	32.9 ± 10.2	84.8 ± 4.0	43.6 ± 11.5
2	**5c**	6-Cl	35.5 ± 1.1	45.3 ± 1.0	86.3 ± 3.3	63.2 ± 13.1
3	**5e**	6-Br	32.9 ± 9.6	40.7 ± 0.1	>100	>100
4	**SGI-1776**		0.03 ± 0.003	4.4 ± 1.7	11.3 ± 4.2	4.0 ± 1.1

^1^ Values are a mean of n ≥ 3 independent experiments. ^2^ Cells were treated with concentrations ranging from 100 nM to 100 μM for 48 h. Cell viability was then determined by MTT assays, and EC_50_ values were calculated using Graphpad PRISM 7 software (n = 3 in triplicate; data are the mean ± SEM).

## Supplementary Materials

The following are available online, spectroscopic data for final compounds, and Figure S1: Inhibition curves of compound **5c** on *Hs*Pim1 (A), *Hs*Pim2 (B), *Rn*DYRK1A (C), and *Hs*GSK3β (D), Figure S2: Inhibition curves of compound **5e** on *Hs*Pim1 (A), *Hs*Pim2 (B), *Rn*DYRK1A (C), and *Hs*GSK3β (D).
