# Supplementary material for: New Quinoxaline Derivatives as Dual Pim-1/2 Kinase Inhibitors: Design, Synthesis and Biological Evaluation
Source: Molecules. 2021 Feb 6;26(4):867. doi: 10.3390/molecules26040867 (PMC7914722; doi:10.3390/molecules26040867)
Supplement: Supplementary file 1 [file molecules-26-00867-s001.pdf]

New quinoxaline derivatives as dual Pim-1/2 kinase inhibitors: design, synthesis and biological evaluation

Bruno Oyallon [1], Marie Brachet-Botineau [2], Cédric Logé [3], Thomas Robert [4,5], Stéphane Bach [4,5,6], Sajida Ibrahim [7], William Raoul [7,8], Cécile Croix [1], Pascal Berthelot [9], Jean Guillon[10], Noël Pinaud [11], Fabrice Gouilleux [2], Marie-Claude Viaud-Massuard [1], Caroline Denevault-Sabourin [1,*]

[1] EA GICC - ERL 7001 CNRS « Groupe Innovation et Ciblage Cellulaire », Team « Innovation Moléculaire et Thérapeutique », University of Tours, F-37200 Tours, France; caroline.denevault@univ-tours.fr

[2] CNRS ERL7001 LNOx « Leukemic Niche and redOx metabolism » - EA GICC, University of Tours, F-37000 Tours, France; fabrice.gouilleux@univ-tours.fr

[3] Université de Nantes, Nantes Atlantique Universités, Département de Chimie Thérapeutique, Cibles et Médicaments des Infections et du Cancer, IICIMED- EA1155, Institut de Recherche en Santé 2, F-44200 Nantes, France; cedric.loge@univ-nantes.fr

[4] Sorbonne Université, CNRS, UMR8227, Integrative Biology of Marine Models Laboratory (LBI2M), Station Biologique de Roscoff, F-29680 Roscoff, France; bach@sb-roscoff.fr

[5] Sorbonne Université, CNRS, FR2424, Plateforme de criblage KISSf (Kinase Inhibitor Specialized Screening facility), Station Biologique de Roscoff, F-29680 Roscoff Cedex, France; trobert@sb-roscoff.fr

[6] Centre of Excellence for Pharmaceutical Sciences, North-West University, Private Bag X6001, Potchefstroom 2520, South Africa

[7] EA GICC - ERL 7001 CNRS « Groupe Innovation et Ciblage Cellulaire », Team « Pharmacologie des Anticorps Thérapeutiques Chez l'Homme », University of Tours, F-37200 Tours, France; william.raoul@univ-tours.fr

[8.] N2C UMR 1069, University of Tours, Inserm, F-37032 Tours, France

[9] UMR-S 1172 - JPArc - Centre de Recherche Jean-Pierre AUBERT Neurosciences et Cancer, University of Lille, Inserm, CHU Lille, F-59000 Lille, France

[10] University of Bordeaux, INSERM U12132 - UMR CNRS 5320, ARNA Laboratory, F-33076 Bordeaux, France; jean.guillon@u-bordeaux.fr

[11] University of Bordeaux, ISM—CNRS UMR 5255, F-33405 Talence, France; noel.pinaud@u-bordeaux.fr

* Correspondence: caroline.denevault@univ-tours.fr; Tel.: +33 2 47-36-72-31 (C.D.S.)

1. NMR spectra……………………………………………………………………………2
2. Mammalian protein kinase assays…………………………………………………22
   2.1. Inhibition curves of compound **5c**……………………………………………22
   2.2. Inhibition curves of compound **5e**……………………………………………23

1. NMR spectra

^{1}H NMR of compound **5a**

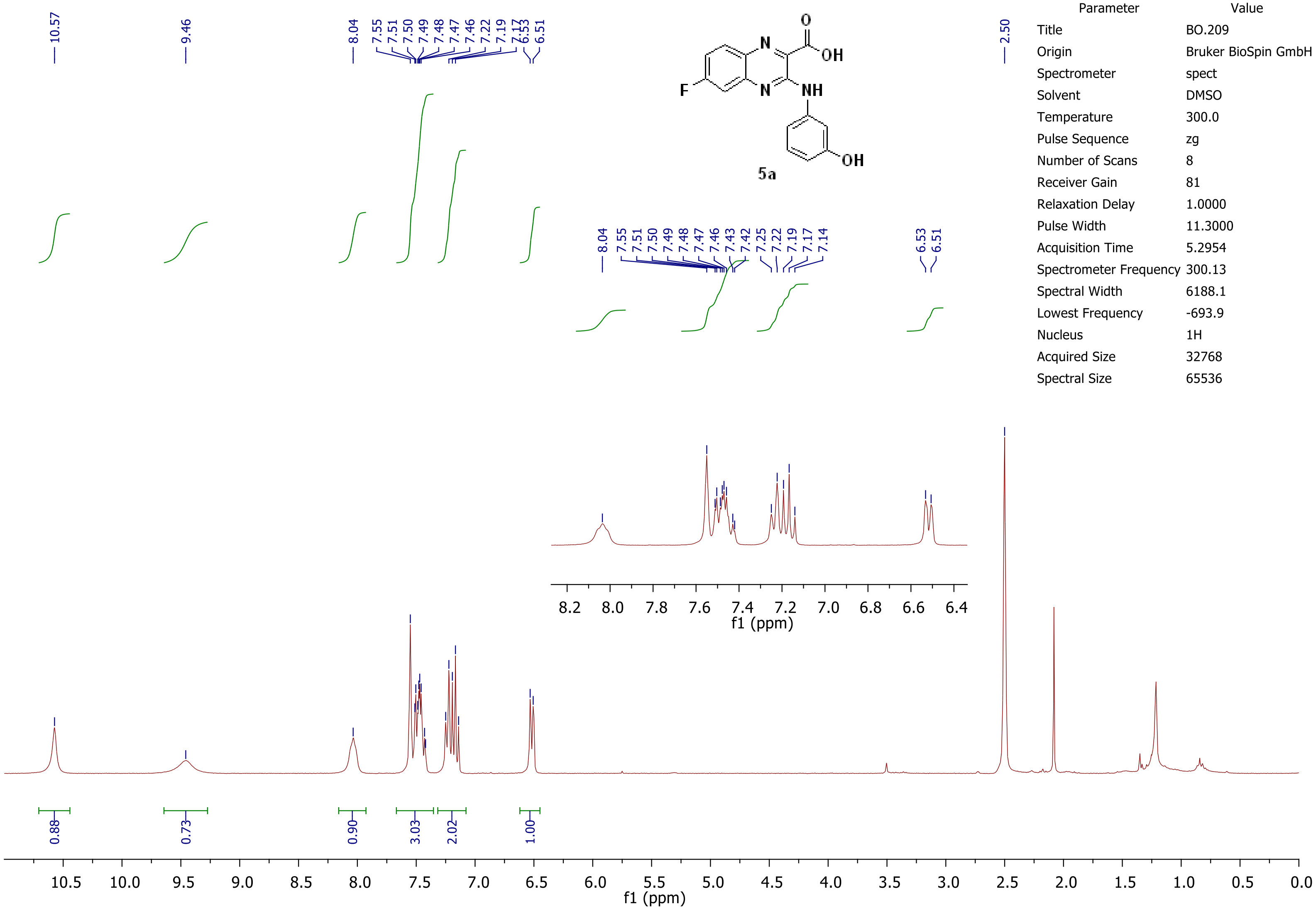

Parameter	Value
Title	BO.209
Origin	Bruker BioSpin GmbH
Spectrometer	spect
Solvent	DMSO
Temperature	300.0
Pulse Sequence	zg
Number of Scans	8
Receiver Gain	81
Relaxation Delay	1.0000
Pulse Width	11.3000
Acquisition Time	5.2954
Spectrometer Frequency	300.13
Spectral Width	6188.1
Lowest Frequency	-693.9
Nucleus	1H
Acquired Size	32768
Spectral Size	65536

^{13}C NMR of compound **5a**

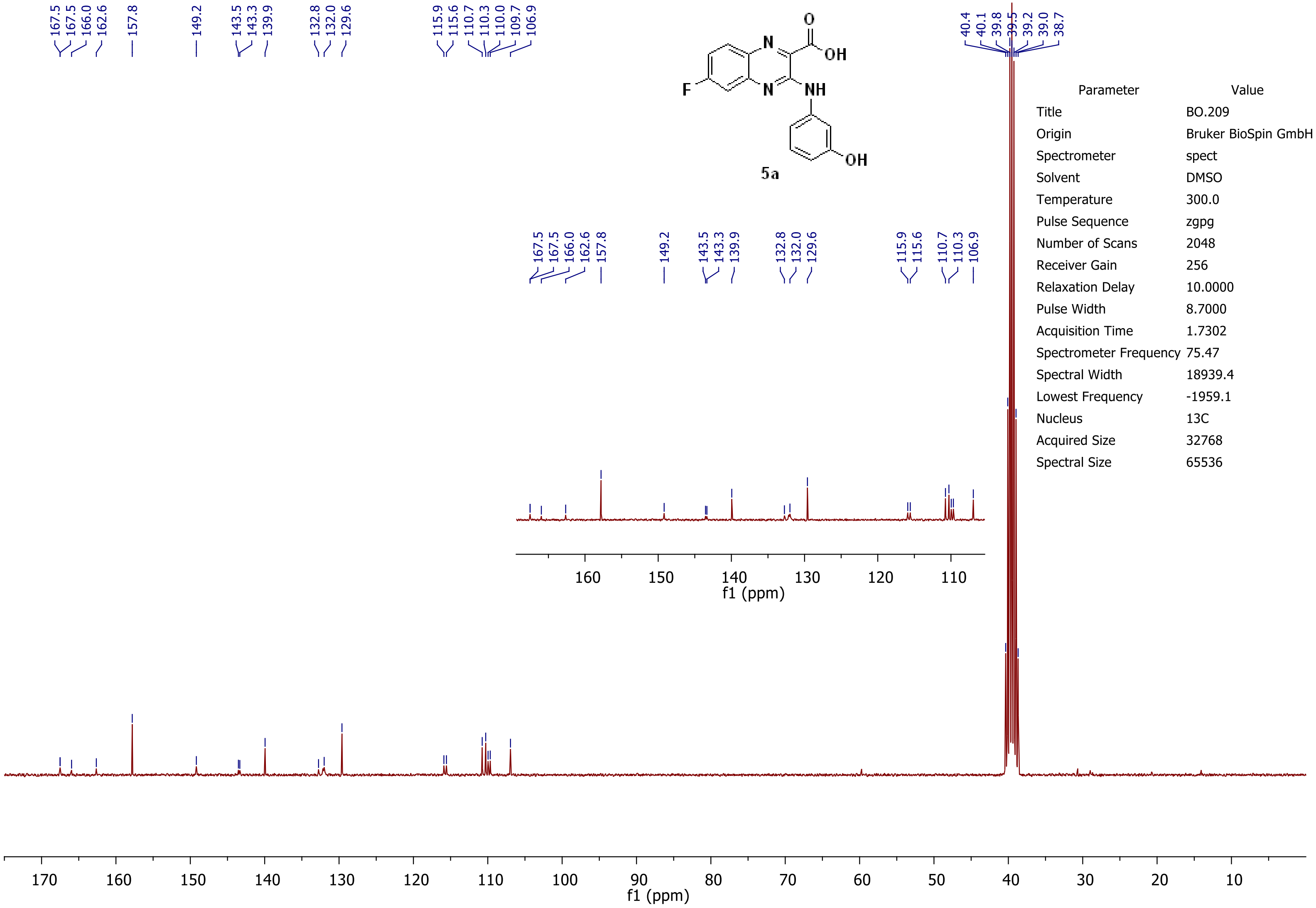

5a

Parameter	Value
Title	BO.209
Origin	Bruker BioSpin GmbH
Spectrometer	spect
Solvent	DMSO
Temperature	300.0
Pulse Sequence	zgpg
Number of Scans	2048
Receiver Gain	256
Relaxation Delay	10.0000
Pulse Width	8.7000
Acquisition Time	1.7302
Spectrometer Frequency	75.47
Spectral Width	18939.4
Lowest Frequency	-1959.1
Nucleus	13C
Acquired Size	32768
Spectral Size	65536

^{19}F NMR of compound **5a**

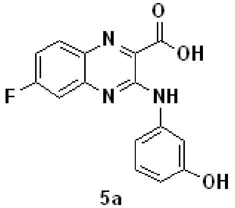

5a

Parameter	Value
Title	BO.209
Origin	Bruker BioSpin GmbH
Spectrometer	spect
Solvent	DMSO
Temperature	300.0
Pulse Sequence	zgfhigqn
Number of Scans	16
Receiver Gain	2050
Relaxation Delay	1.0000
Pulse Width	8.6000
Acquisition Time	0.5768
Spectrometer Frequency	282.40
Spectral Width	113636.4
Lowest Frequency	-85058.4
Nucleus	19F
Acquired Size	65536
Spectral Size	131072

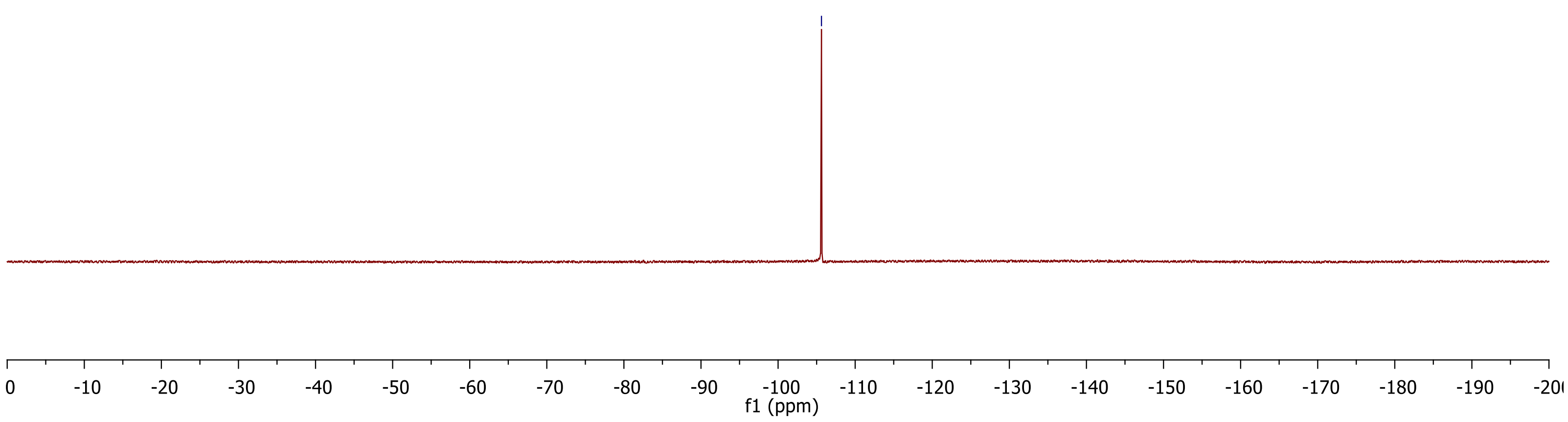

^{1}H NMR of compound **5b**

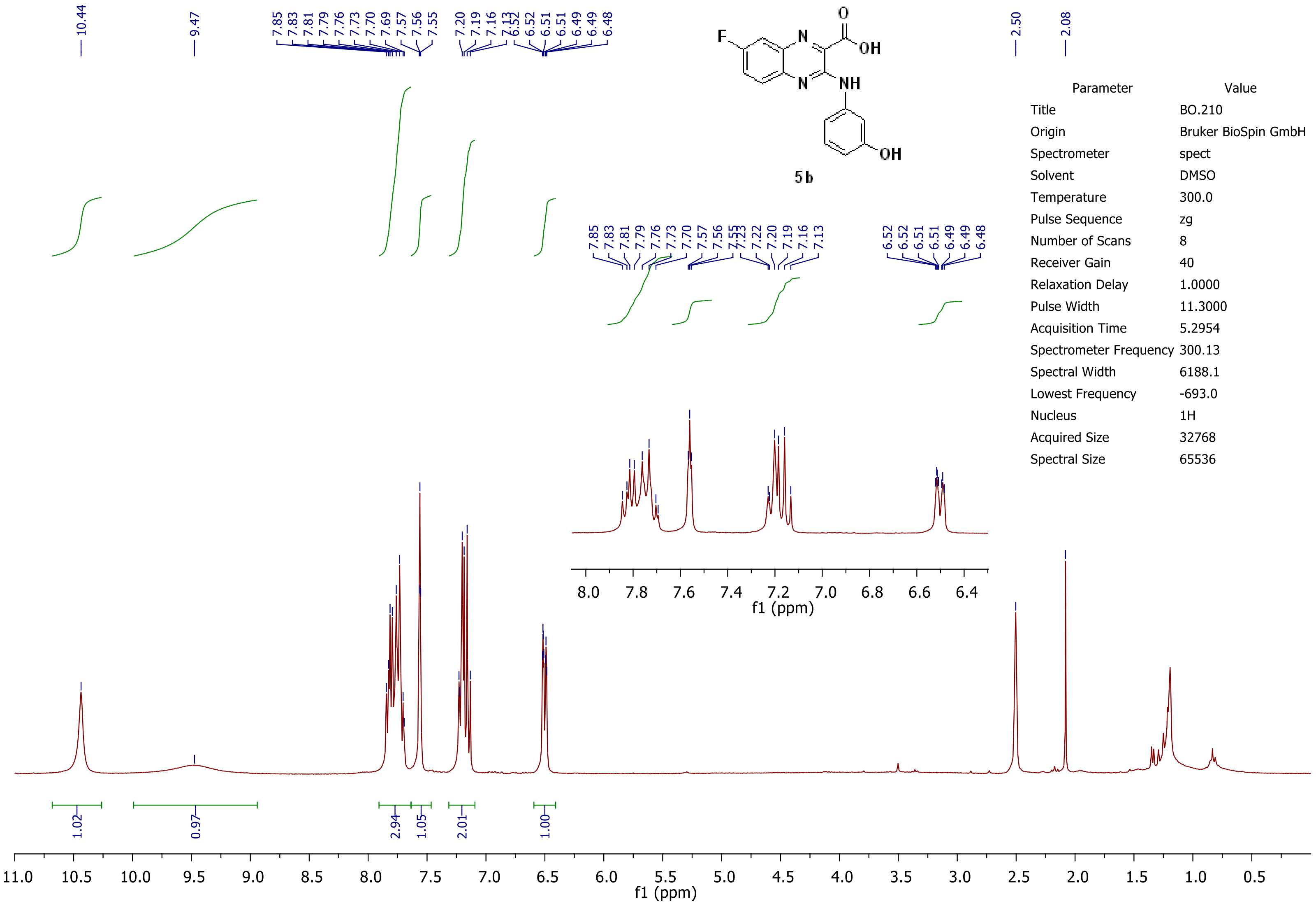

5b

Parameter	Value
Title	BO.210
Origin	Bruker BioSpin GmbH
Spectrometer	spect
Solvent	DMSO
Temperature	300.0
Pulse Sequence	zg
Number of Scans	8
Receiver Gain	40
Relaxation Delay	1.0000
Pulse Width	11.3000
Acquisition Time	5.2954
Spectrometer Frequency	300.13
Spectral Width	6188.1
Lowest Frequency	-693.0
Nucleus	1H
Acquired Size	32768
Spectral Size	65536

^{13}C NMR of compound **5b**

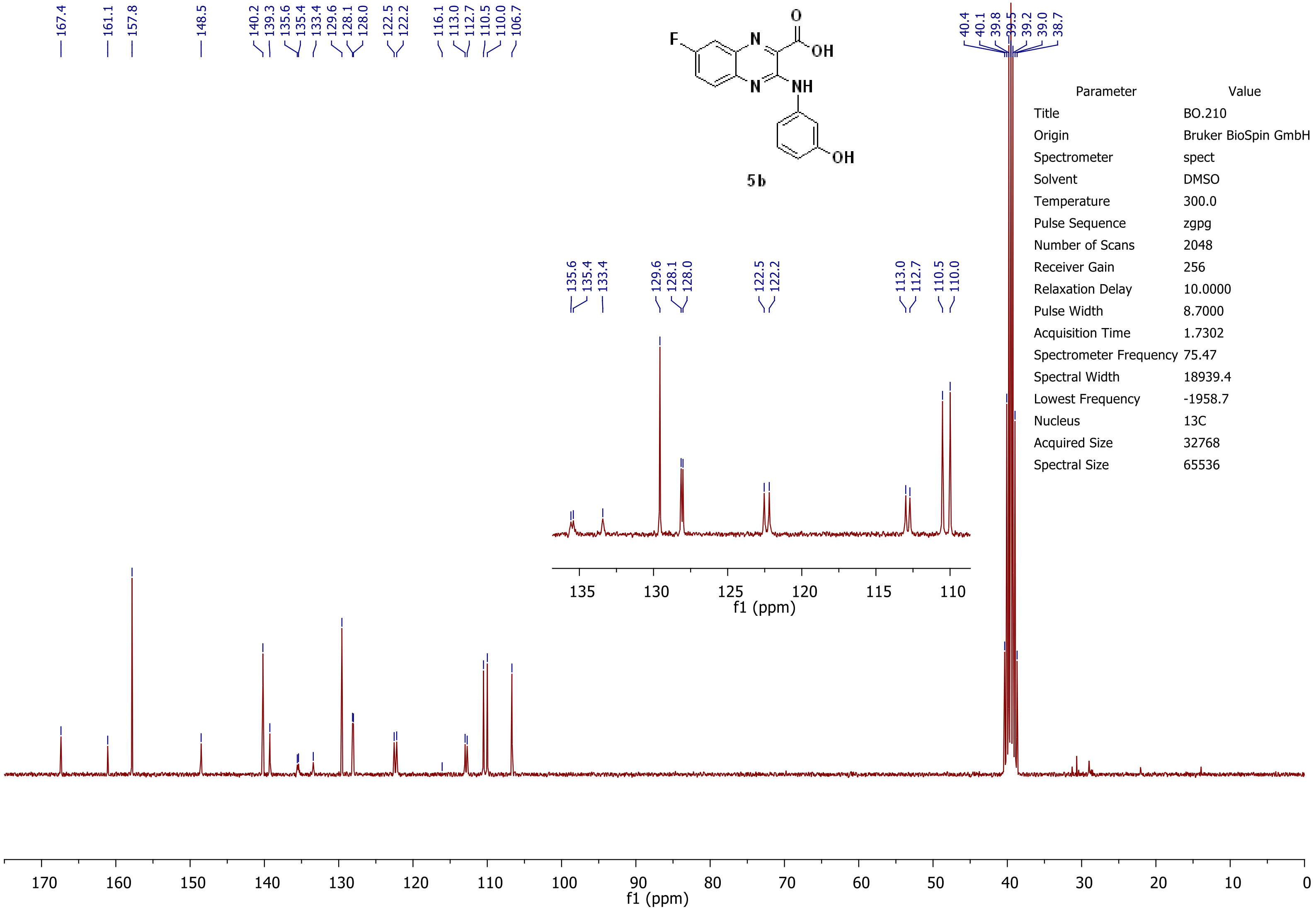

Parameter	Value
Title	BO.210
Origin	Bruker BioSpin GmbH
Spectrometer	spect
Solvent	DMSO
Temperature	300.0
Pulse Sequence	zgpg
Number of Scans	2048
Receiver Gain	256
Relaxation Delay	10.0000
Pulse Width	8.7000
Acquisition Time	1.7302
Spectrometer Frequency	75.47
Spectral Width	18939.4
Lowest Frequency	-1958.7
Nucleus	13C
Acquired Size	32768
Spectral Size	65536

^{19}F NMR of compound **5b**

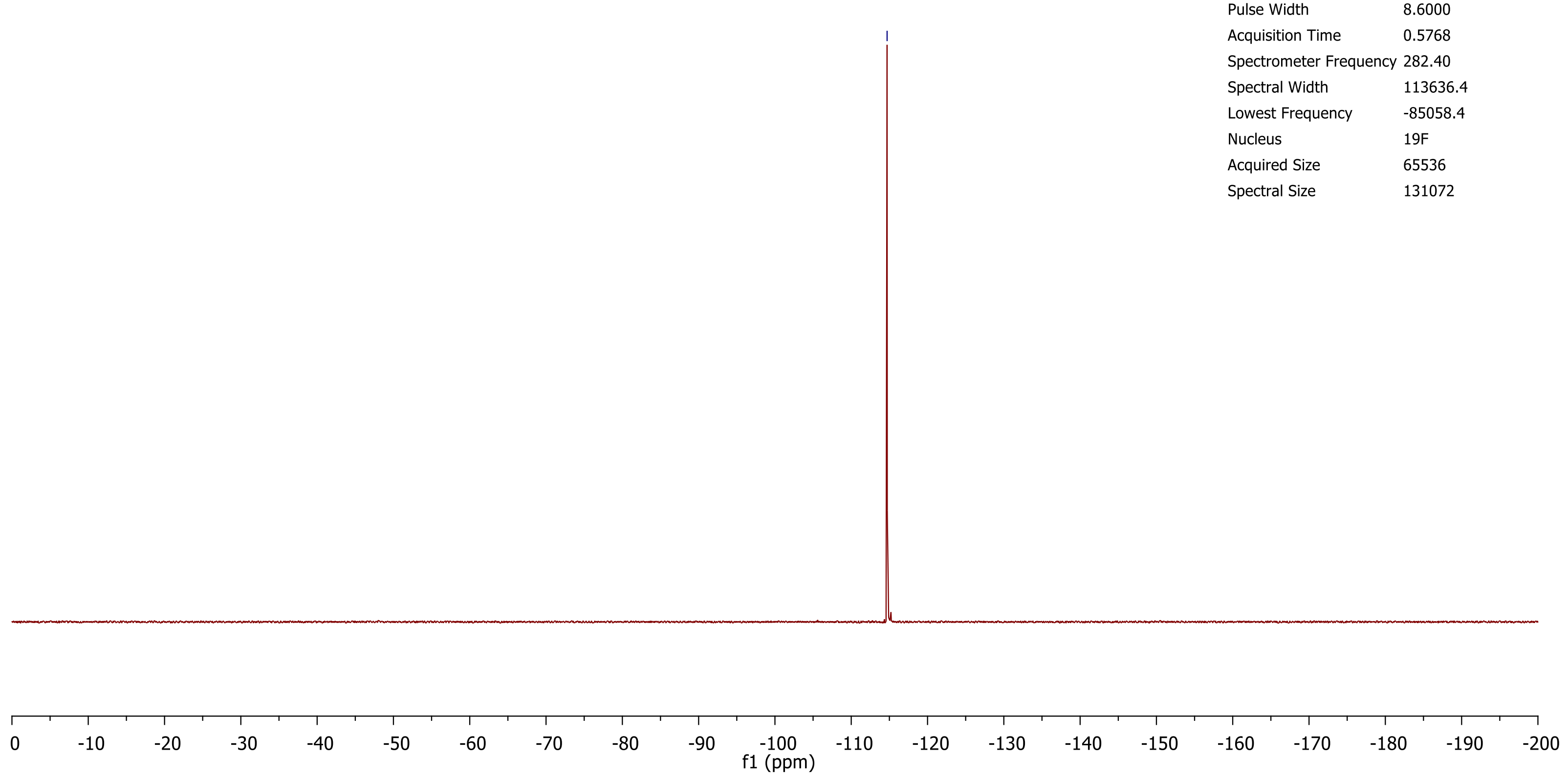

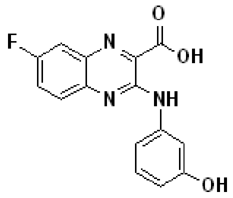

5b

Parameter	Value
Title	BO.210
Origin	Bruker BioSpin GmbH
Spectrometer	spect
Solvent	DMSO
Temperature	300.0
Pulse Sequence	zgfhigqn
Number of Scans	16
Receiver Gain	2050
Relaxation Delay	1.0000
Pulse Width	8.6000
Acquisition Time	0.5768
Spectrometer Frequency	282.40
Spectral Width	113636.4
Lowest Frequency	-85058.4
Nucleus	19F
Acquired Size	65536
Spectral Size	131072

^{1}H NMR of compound **5c**

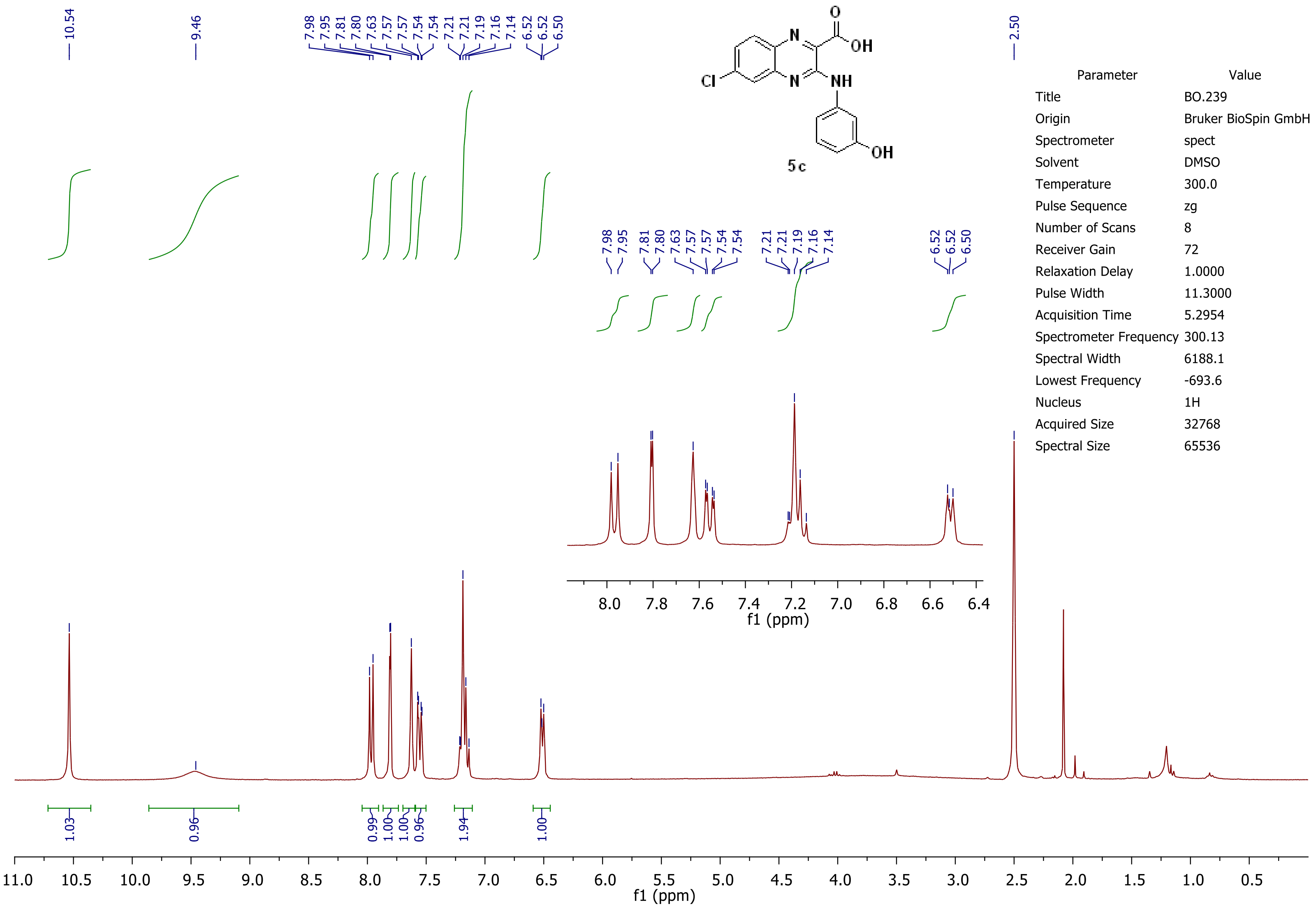

Parameter	Value
Title	BO.239
Origin	Bruker BioSpin GmbH
Spectrometer	spect
Solvent	DMSO
Temperature	300.0
Pulse Sequence	zg
Number of Scans	8
Receiver Gain	72
Relaxation Delay	1.0000
Pulse Width	11.3000
Acquisition Time	5.2954
Spectrometer Frequency	300.13
Spectral Width	6188.1
Lowest Frequency	-693.6
Nucleus	1H
Acquired Size	32768
Spectral Size	65536

^{13}C NMR of compound **5c**

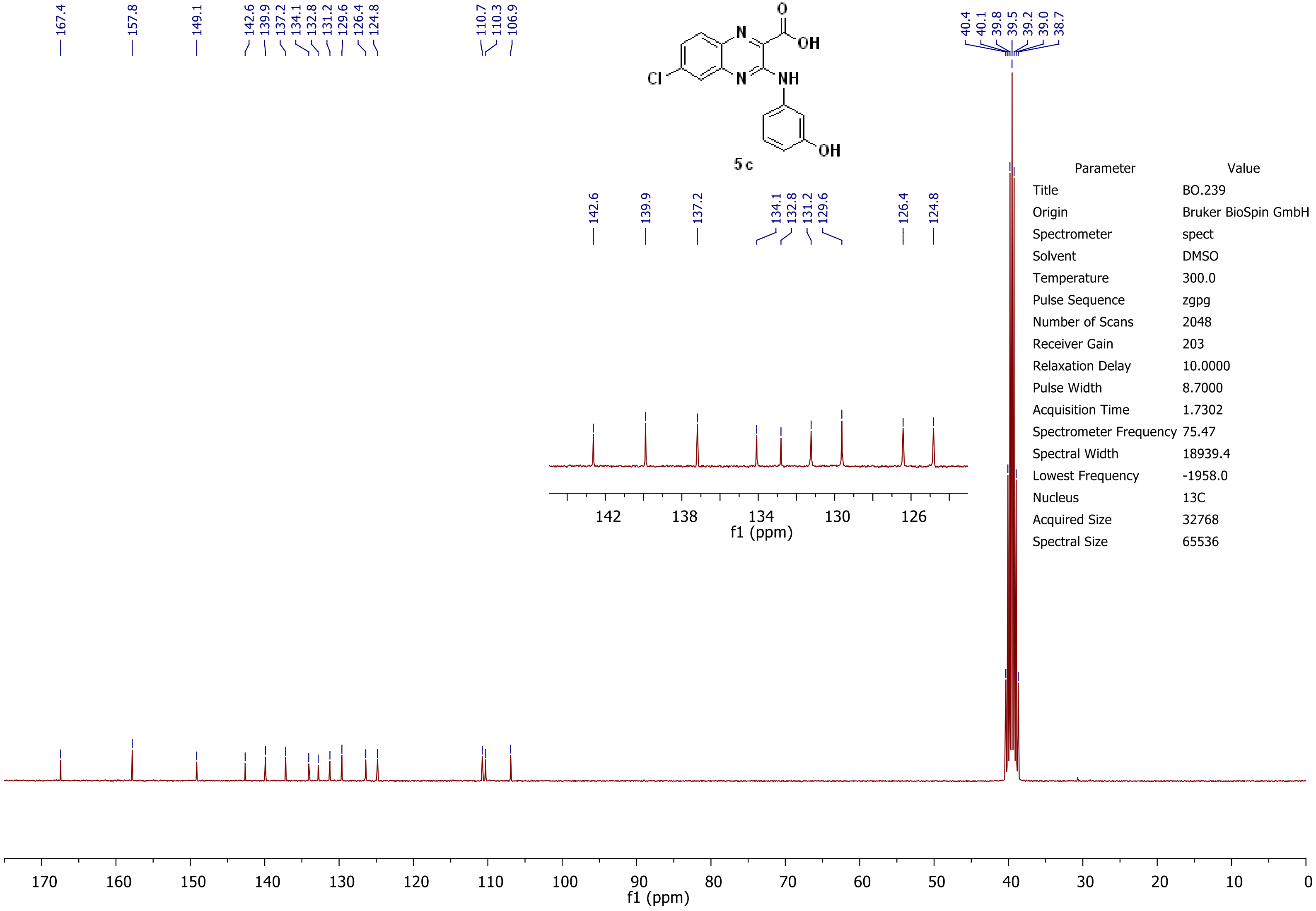

Parameter	Value
Title	BO.239
Origin	Bruker BioSpin GmbH
Spectrometer	spect
Solvent	DMSO
Temperature	300.0
Pulse Sequence	zgpg
Number of Scans	2048
Receiver Gain	203
Relaxation Delay	10.0000
Pulse Width	8.7000
Acquisition Time	1.7302
Spectrometer Frequency	75.47
Spectral Width	18939.4
Lowest Frequency	-1958.0
Nucleus	13C
Acquired Size	32768
Spectral Size	65536

^{1}H NMR of compound **5d**

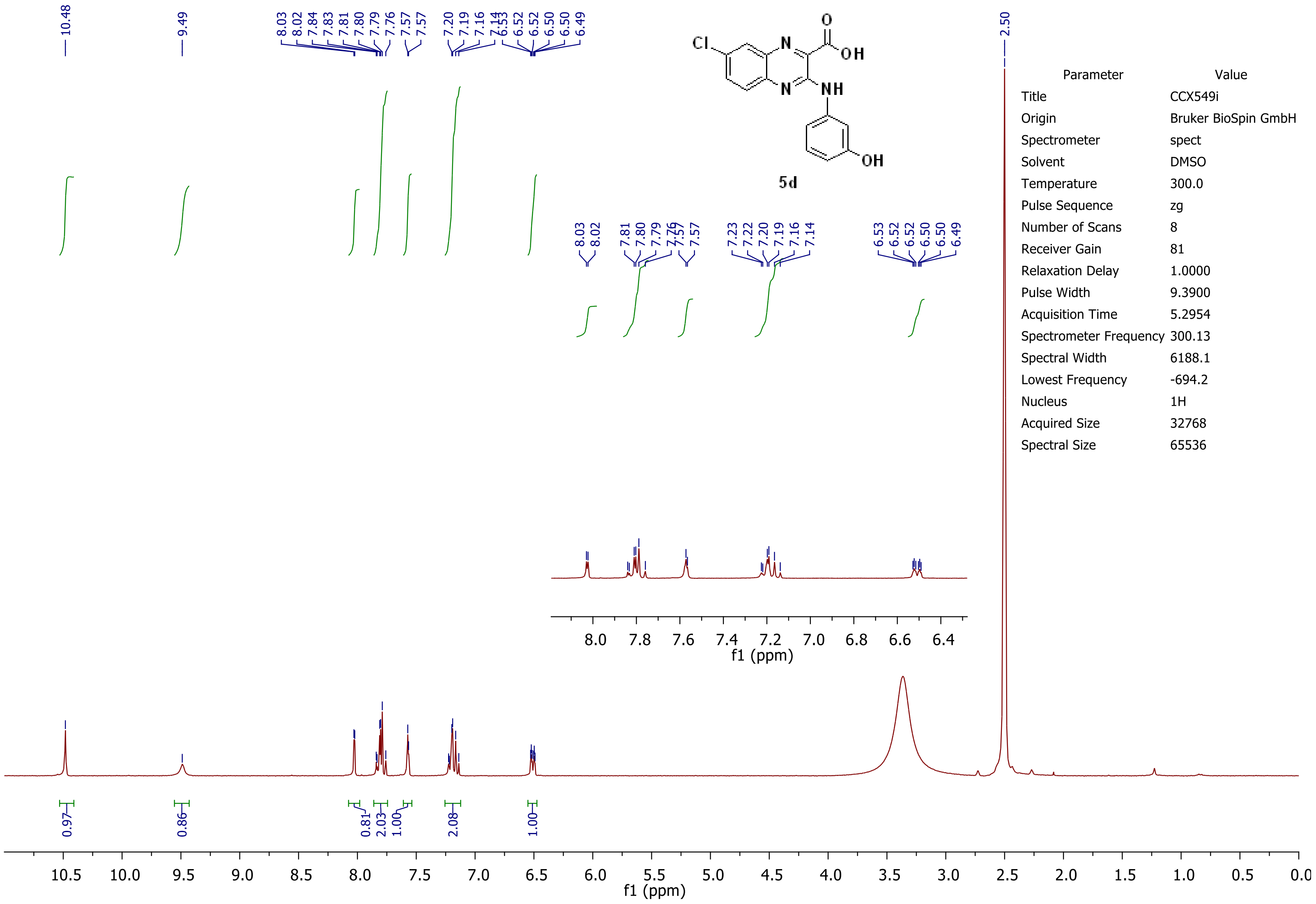

Parameter	Value
Title	CCX549i
Origin	Bruker BioSpin GmbH
Spectrometer	spect
Solvent	DMSO
Temperature	300.0
Pulse Sequence	zg
Number of Scans	8
Receiver Gain	81
Relaxation Delay	1.0000
Pulse Width	9.3900
Acquisition Time	5.2954
Spectrometer Frequency	300.13
Spectral Width	6188.1
Lowest Frequency	-694.2
Nucleus	1H
Acquired Size	32768
Spectral Size	65536

^{13}C NMR of compound **5d**

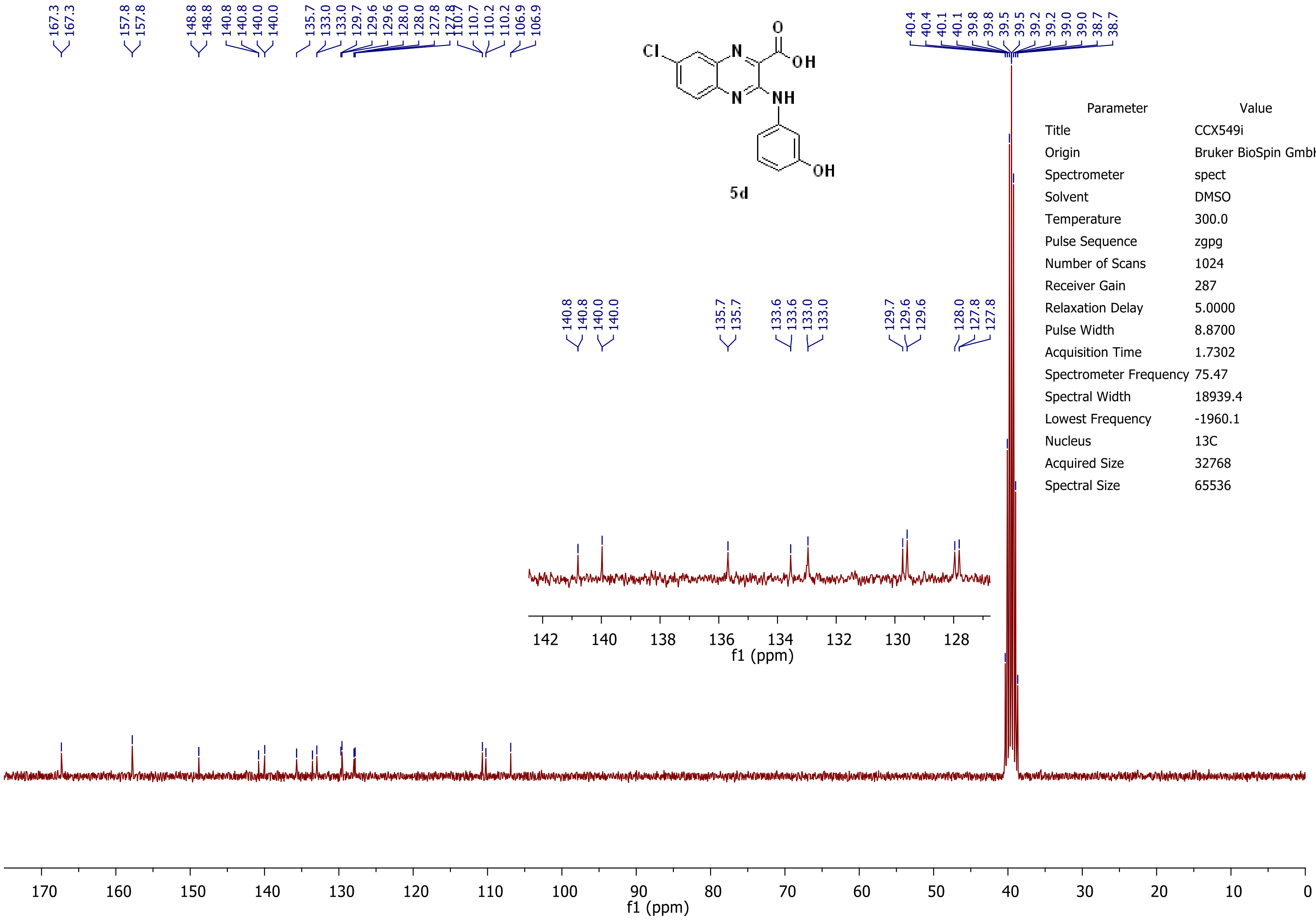

Parameter	Value
Title	CCX549i
Origin	Bruker BioSpin GmbH
Spectrometer	spect
Solvent	DMSO
Temperature	300.0
Pulse Sequence	zgpg
Number of Scans	1024
Receiver Gain	287
Relaxation Delay	5.0000
Pulse Width	8.8700
Acquisition Time	1.7302
Spectrometer Frequency	75.47
Spectral Width	18939.4
Lowest Frequency	-1960.1
Nucleus	13C
Acquired Size	32768
Spectral Size	65536

^{1}H NMR of compound **5e**

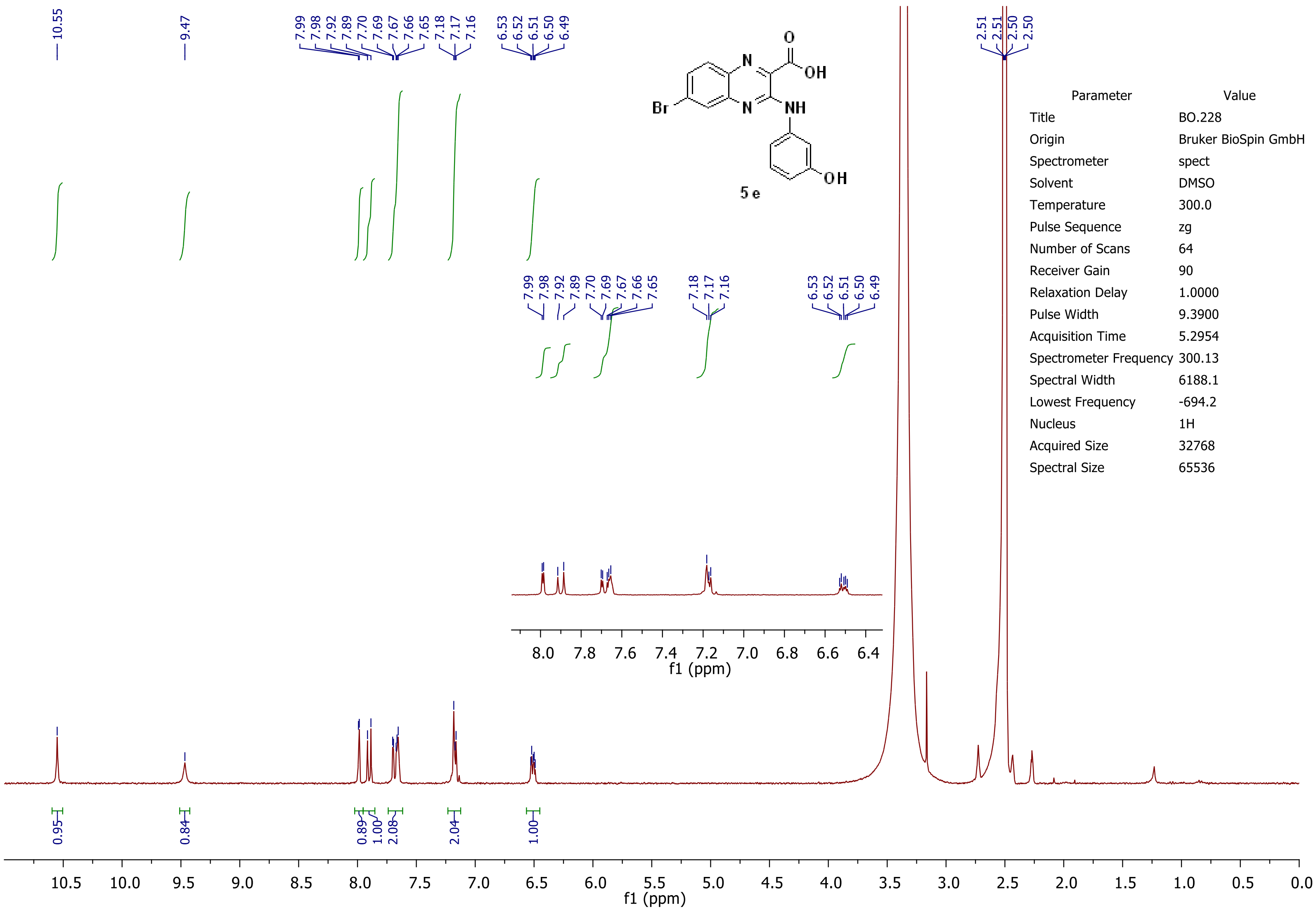

Parameter	Value
Title	BO.228
Origin	Bruker BioSpin GmbH
Spectrometer	spect
Solvent	DMSO
Temperature	300.0
Pulse Sequence	zg
Number of Scans	64
Receiver Gain	90
Relaxation Delay	1.0000
Pulse Width	9.3900
Acquisition Time	5.2954
Spectrometer Frequency	300.13
Spectral Width	6188.1
Lowest Frequency	-694.2
Nucleus	1H
Acquired Size	32768
Spectral Size	65536

^{13}C NMR of compound **5e**

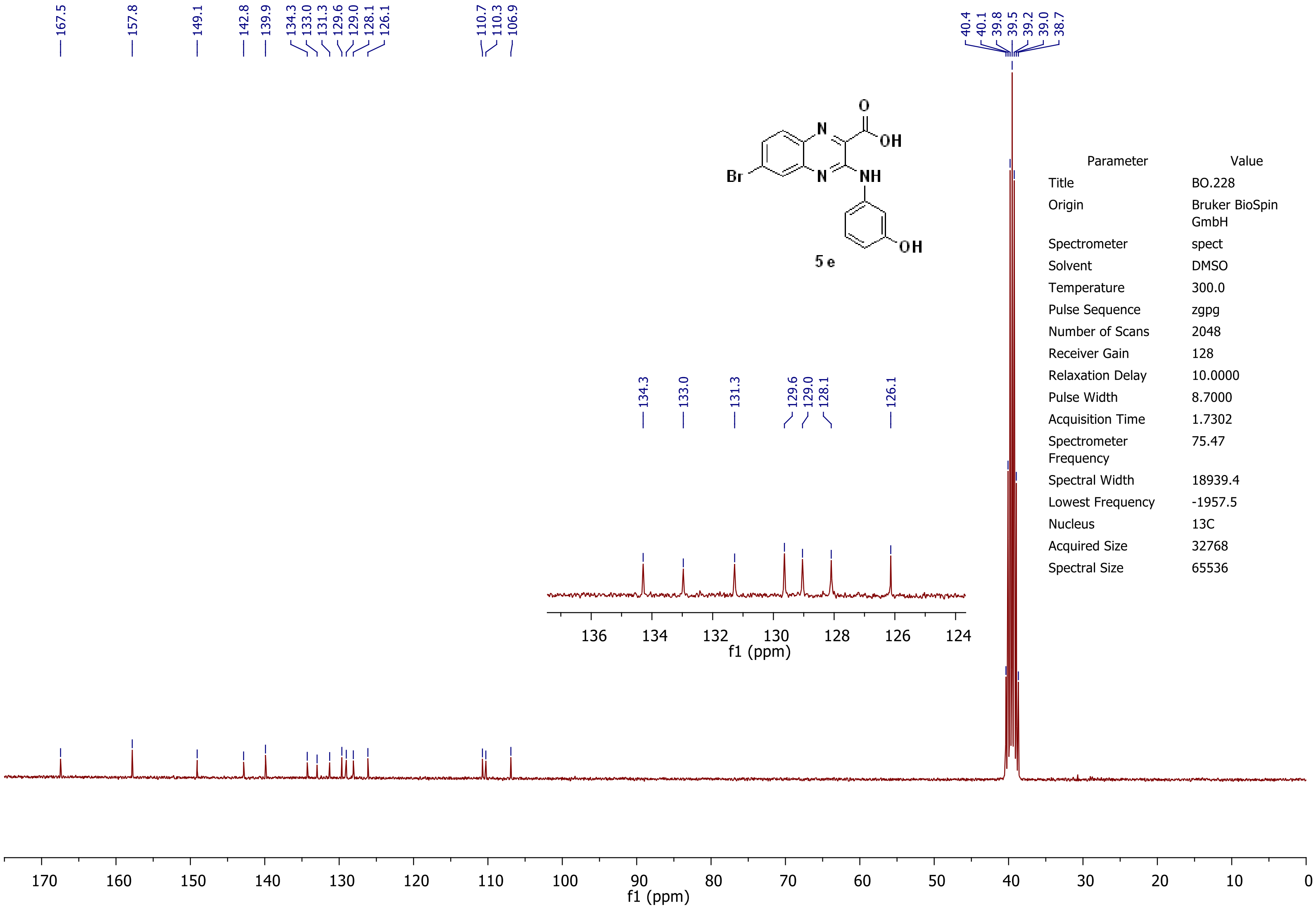

Parameter	Value
Title	BO.228
Origin	Bruker BioSpin GmbH
Spectrometer	spect
Solvent	DMSO
Temperature	300.0
Pulse Sequence	zgpg
Number of Scans	2048
Receiver Gain	128
Relaxation Delay	10.0000
Pulse Width	8.7000
Acquisition Time	1.7302
Spectrometer Frequency	75.47
Spectral Width	18939.4
Lowest Frequency	-1957.5
Nucleus	13C
Acquired Size	32768
Spectral Size	65536

^{1}H NMR of compound **5f**

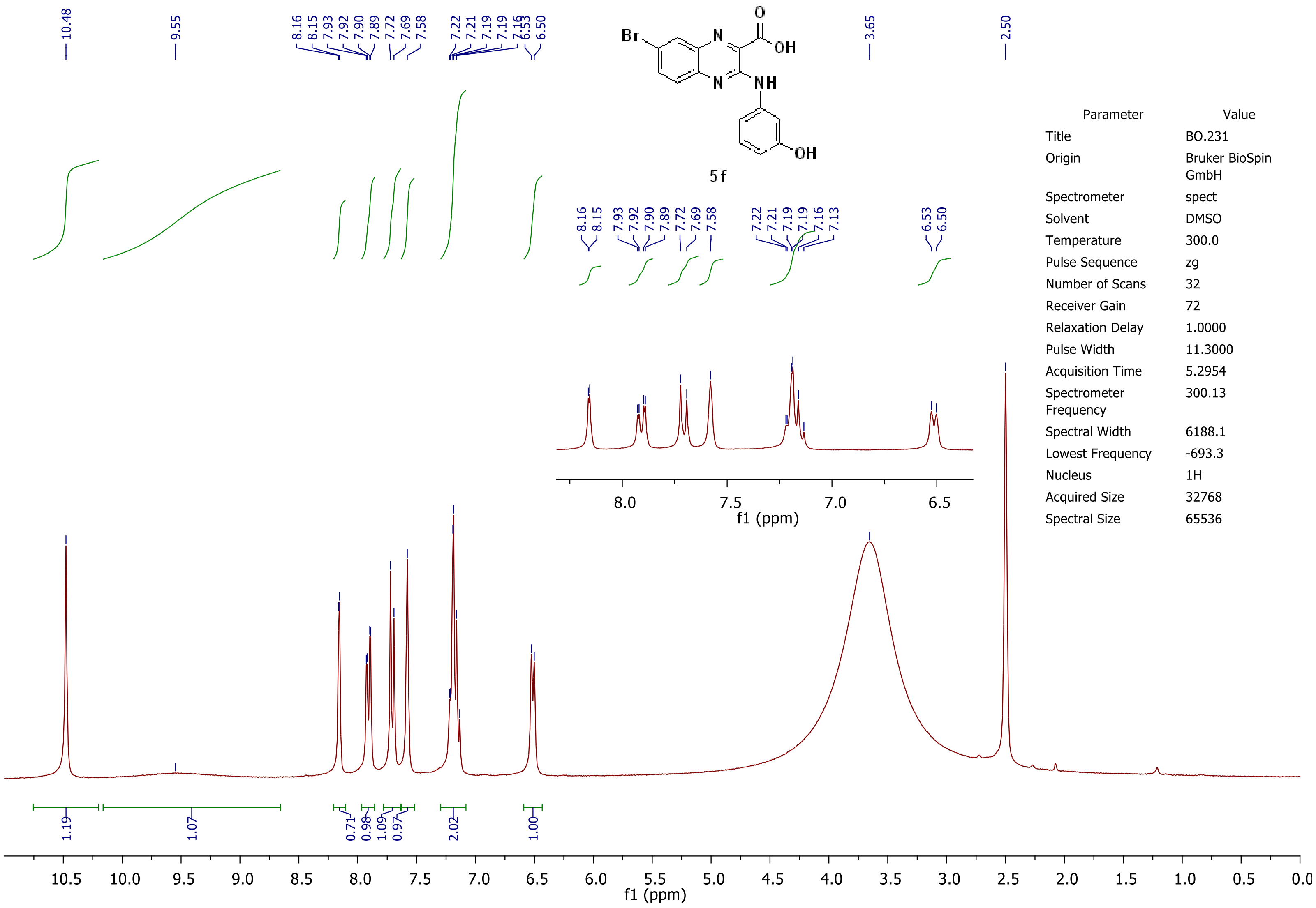

Parameter	Value
Title	BO.231
Origin	Bruker BioSpin GmbH
Spectrometer	spect
Solvent	DMSO
Temperature	300.0
Pulse Sequence	zg
Number of Scans	32
Receiver Gain	72
Relaxation Delay	1.0000
Pulse Width	11.3000
Acquisition Time	5.2954
Spectrometer Frequency	300.13
Spectral Width	6188.1
Lowest Frequency	-693.3
Nucleus	1H
Acquired Size	32768
Spectral Size	65536

^{13}C NMR of compound **5f**

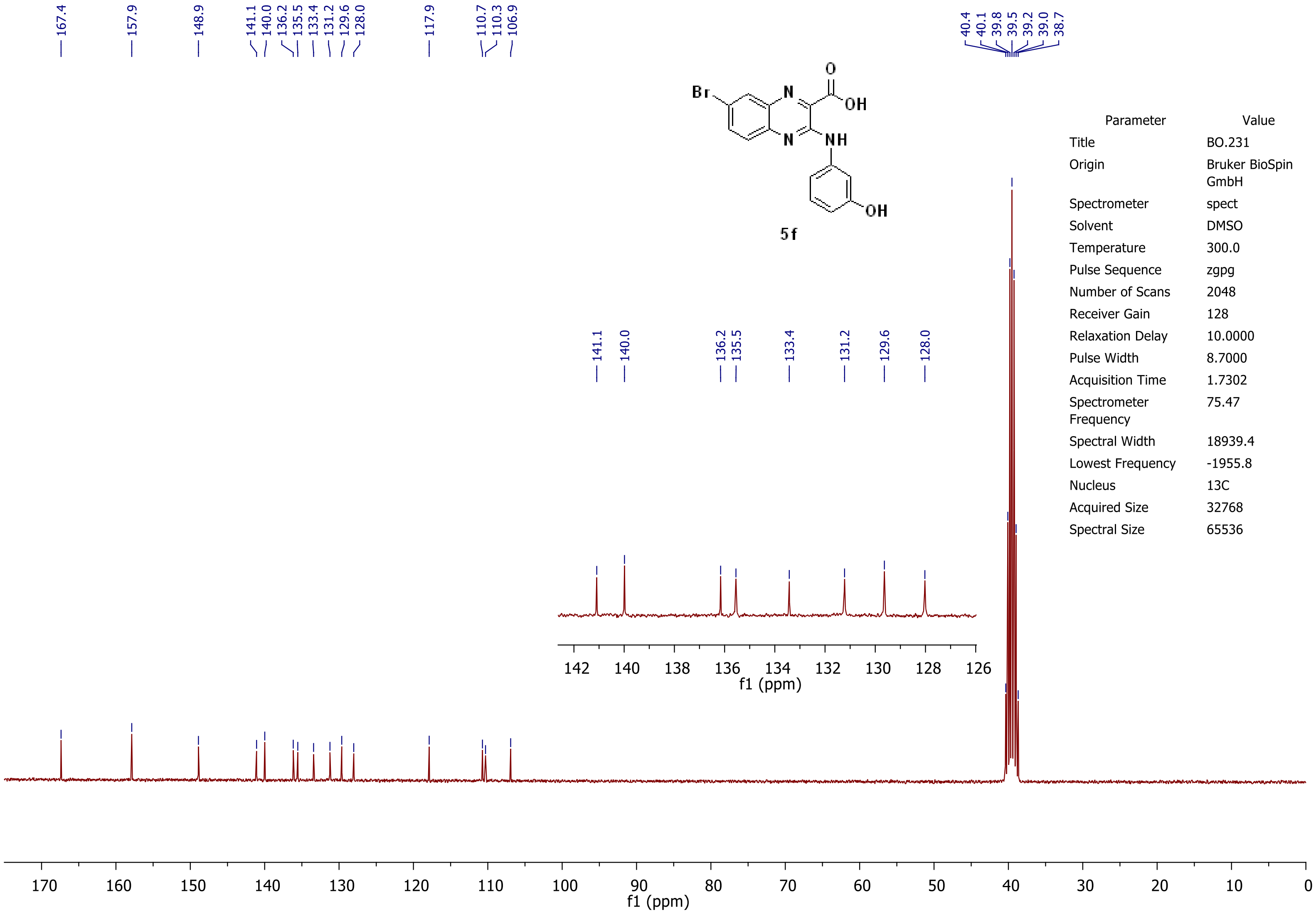

Parameter	Value
Title	BO.231
Origin	Bruker BioSpin GmbH
Spectrometer	spect
Solvent	DMSO
Temperature	300.0
Pulse Sequence	zgpg
Number of Scans	2048
Receiver Gain	128
Relaxation Delay	10.0000
Pulse Width	8.7000
Acquisition Time	1.7302
Spectrometer Frequency	75.47
Spectral Width	18939.4
Lowest Frequency	-1955.8
Nucleus	13C
Acquired Size	32768
Spectral Size	65536

^{1}H NMR of compound **5g**

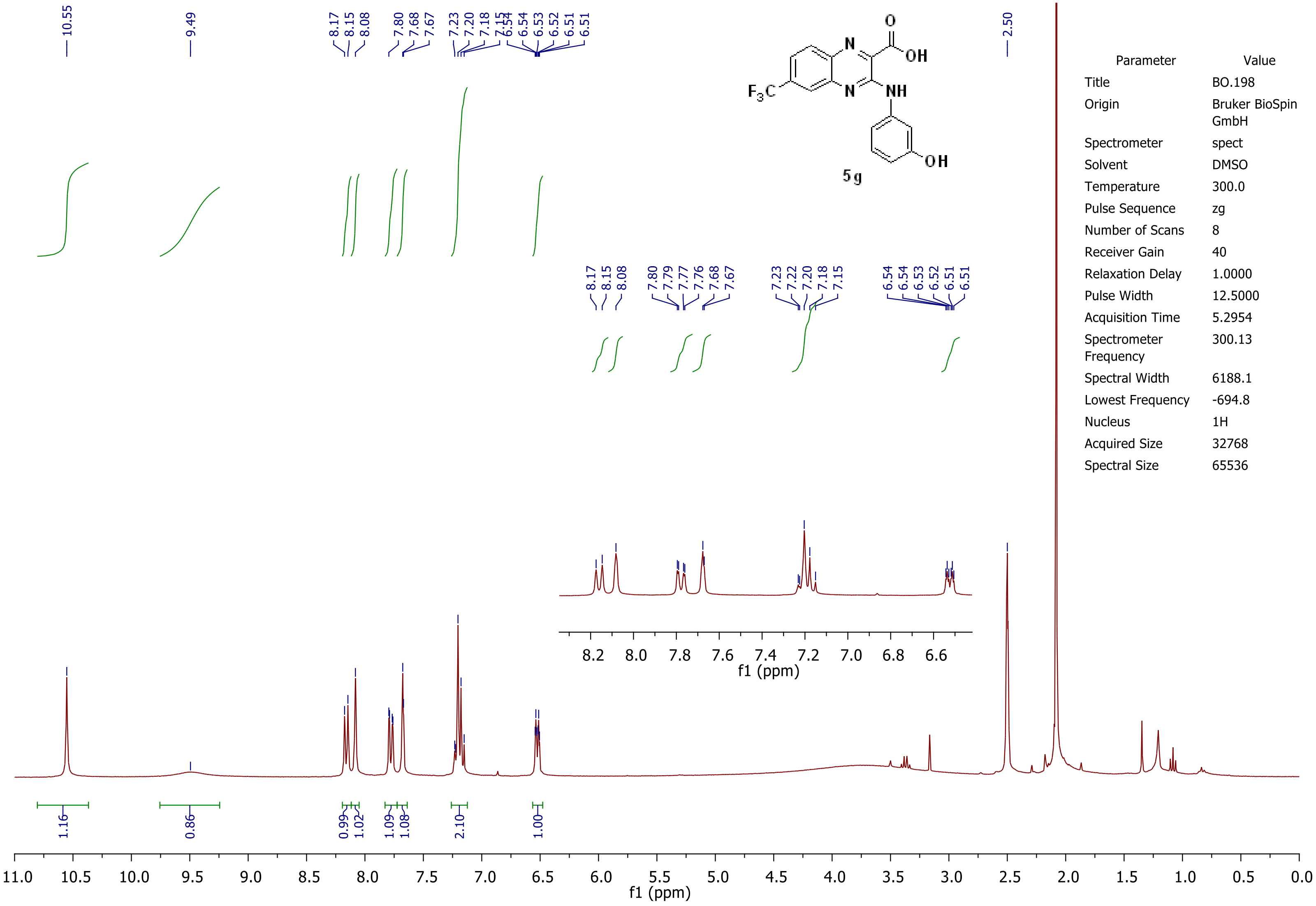

Parameter	Value
Title	BO.198
Origin	Bruker BioSpin GmbH
Spectrometer	spect
Solvent	DMSO
Temperature	300.0
Pulse Sequence	zg
Number of Scans	8
Receiver Gain	40
Relaxation Delay	1.0000
Pulse Width	12.5000
Acquisition Time	5.2954
Spectrometer Frequency	300.13
Spectral Width	6188.1
Lowest Frequency	-694.8
Nucleus	1H
Acquired Size	32768
Spectral Size	65536

^{13}C NMR of compound **5g**

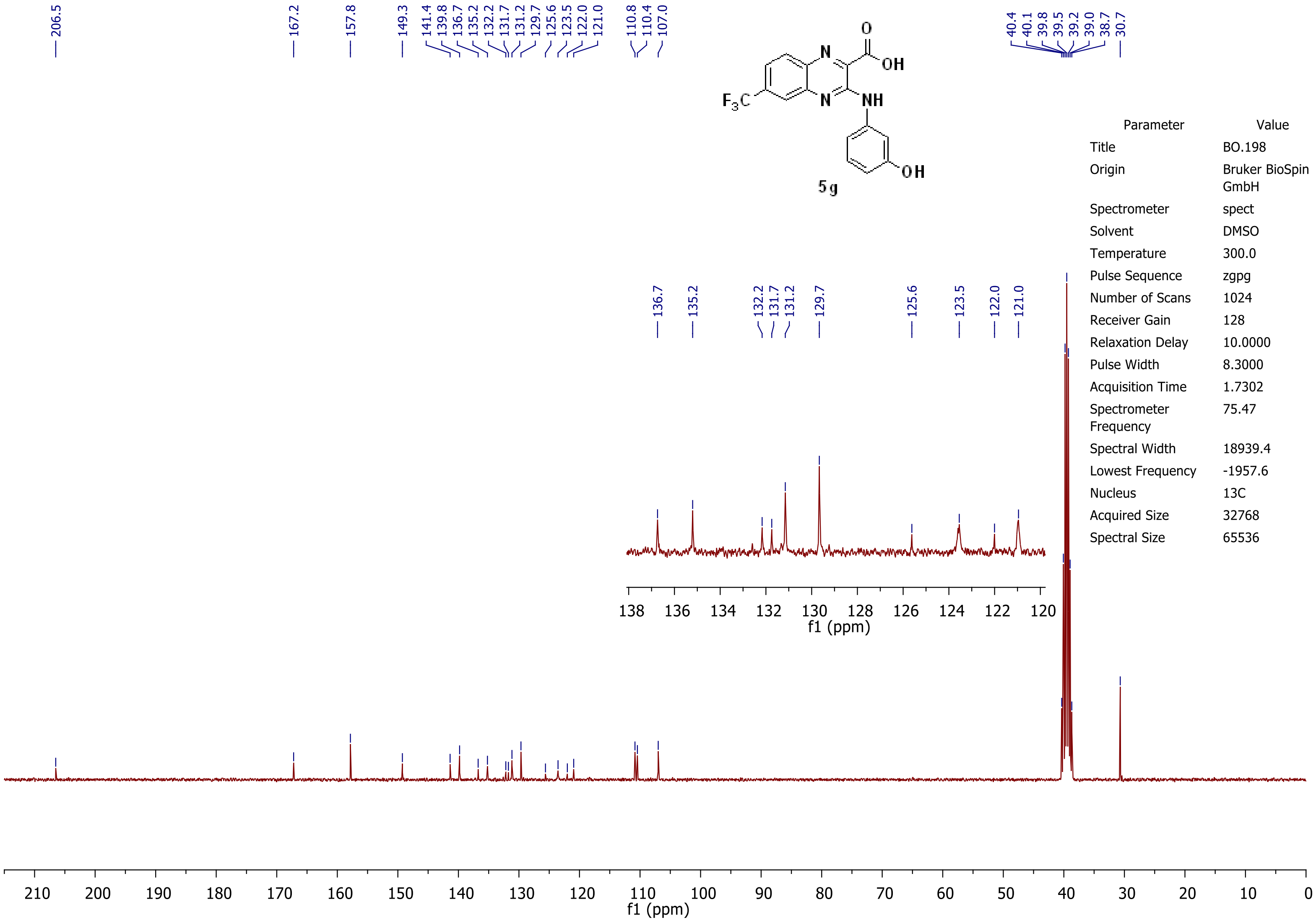

Parameter	Value
Title	BO.198
Origin	Bruker BioSpin GmbH
Spectrometer	spect
Solvent	DMSO
Temperature	300.0
Pulse Sequence	zgpg
Number of Scans	1024
Receiver Gain	128
Relaxation Delay	10.0000
Pulse Width	8.3000
Acquisition Time	1.7302
Spectrometer Frequency	75.47
Spectral Width	18939.4
Lowest Frequency	-1957.6
Nucleus	13C
Acquired Size	32768
Spectral Size	65536

^{19}F NMR of compound **5g**

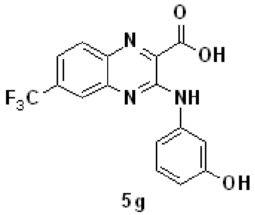

5g

Parameter	Value
Title	BO.198
Origin	Bruker BioSpin GmbH
Spectrometer	spect
Solvent	DMSO
Temperature	300.0
Pulse Sequence	zgfhigqn
Number of Scans	16
Receiver Gain	2050
Relaxation Delay	1.0000
Pulse Width	8.4000
Acquisition Time	0.5768
Spectrometer Frequency	282.40
Spectral Width	113636.4
Lowest Frequency	-85058.4
Nucleus	19F
Acquired Size	65536
Spectral Size	131072

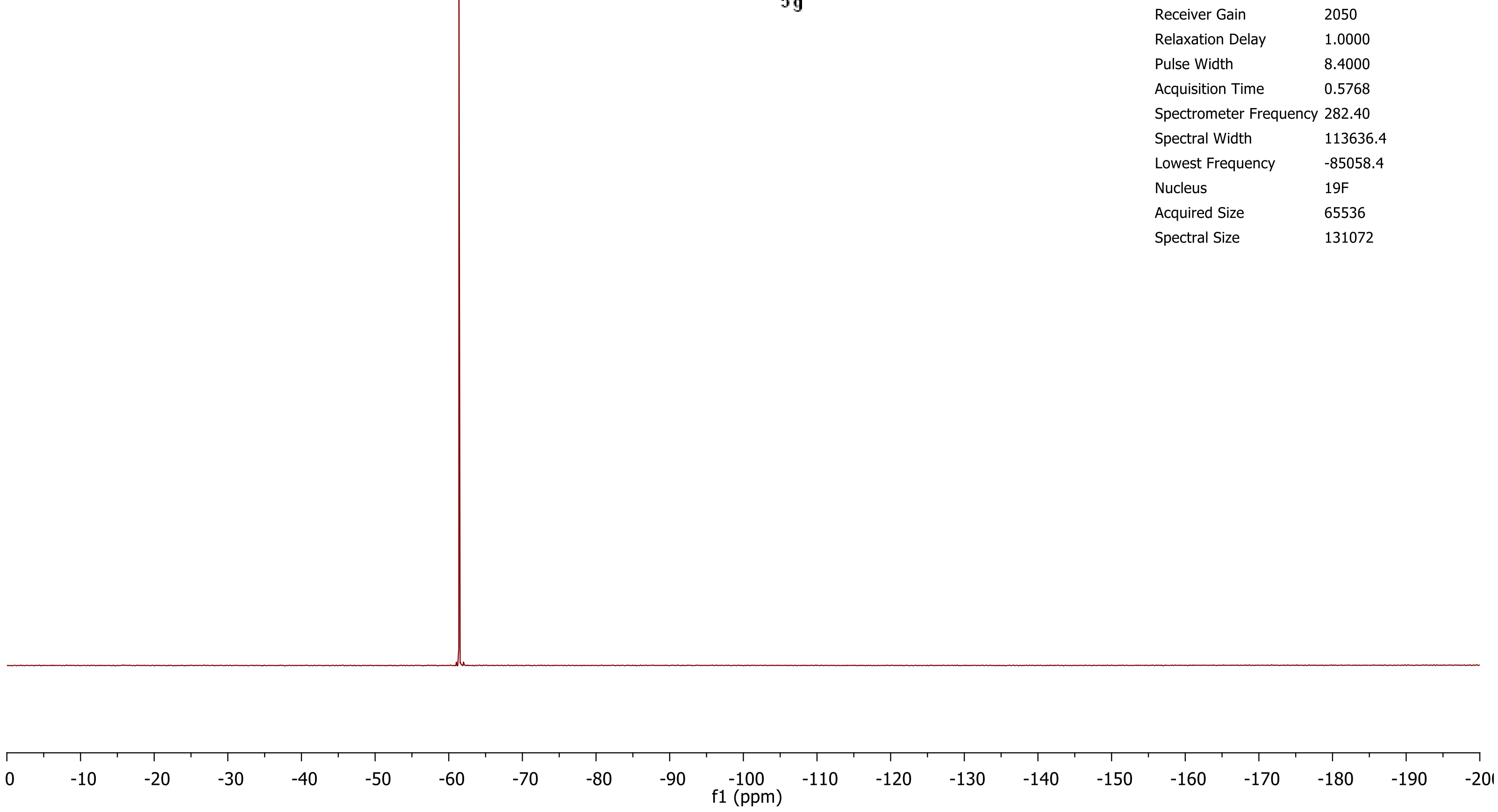

^{1}H NMR of compound **5h**

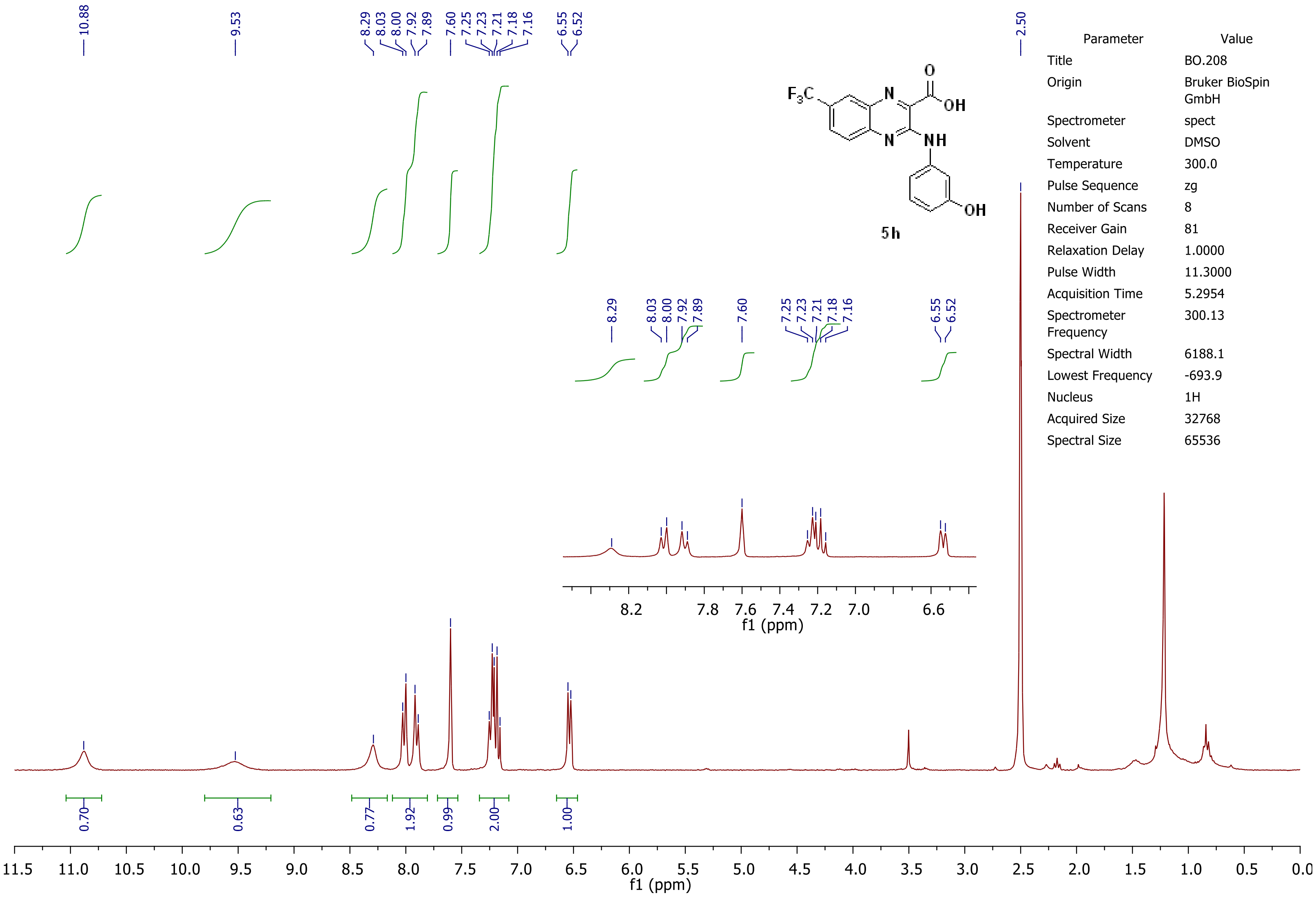

Parameter	Value
Title	BO.208
Origin	Bruker BioSpin GmbH
Spectrometer	spect
Solvent	DMSO
Temperature	300.0
Pulse Sequence	zg
Number of Scans	8
Receiver Gain	81
Relaxation Delay	1.0000
Pulse Width	11.3000
Acquisition Time	5.2954
Spectrometer Frequency	300.13
Spectral Width	6188.1
Lowest Frequency	-693.9
Nucleus	1H
Acquired Size	32768
Spectral Size	65536

^{13}C NMR of compound **5h**

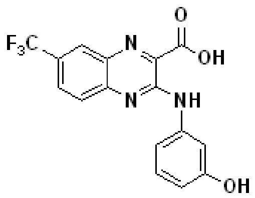

5h

Parameter	Value
Title	BO.208
Origin	Bruker BioSpin GmbH
Spectrometer	spect
Solvent	DMSO
Temperature	300.0
Pulse Sequence	zgpg
Number of Scans	2048
Receiver Gain	203
Relaxation Delay	10.0000
Pulse Width	8.7000
Acquisition Time	1.7302
Spectrometer Frequency	75.47
Spectral Width	18939.4
Lowest Frequency	-1923.4
Nucleus	13C
Acquired Size	32768
Spectral Size	65536

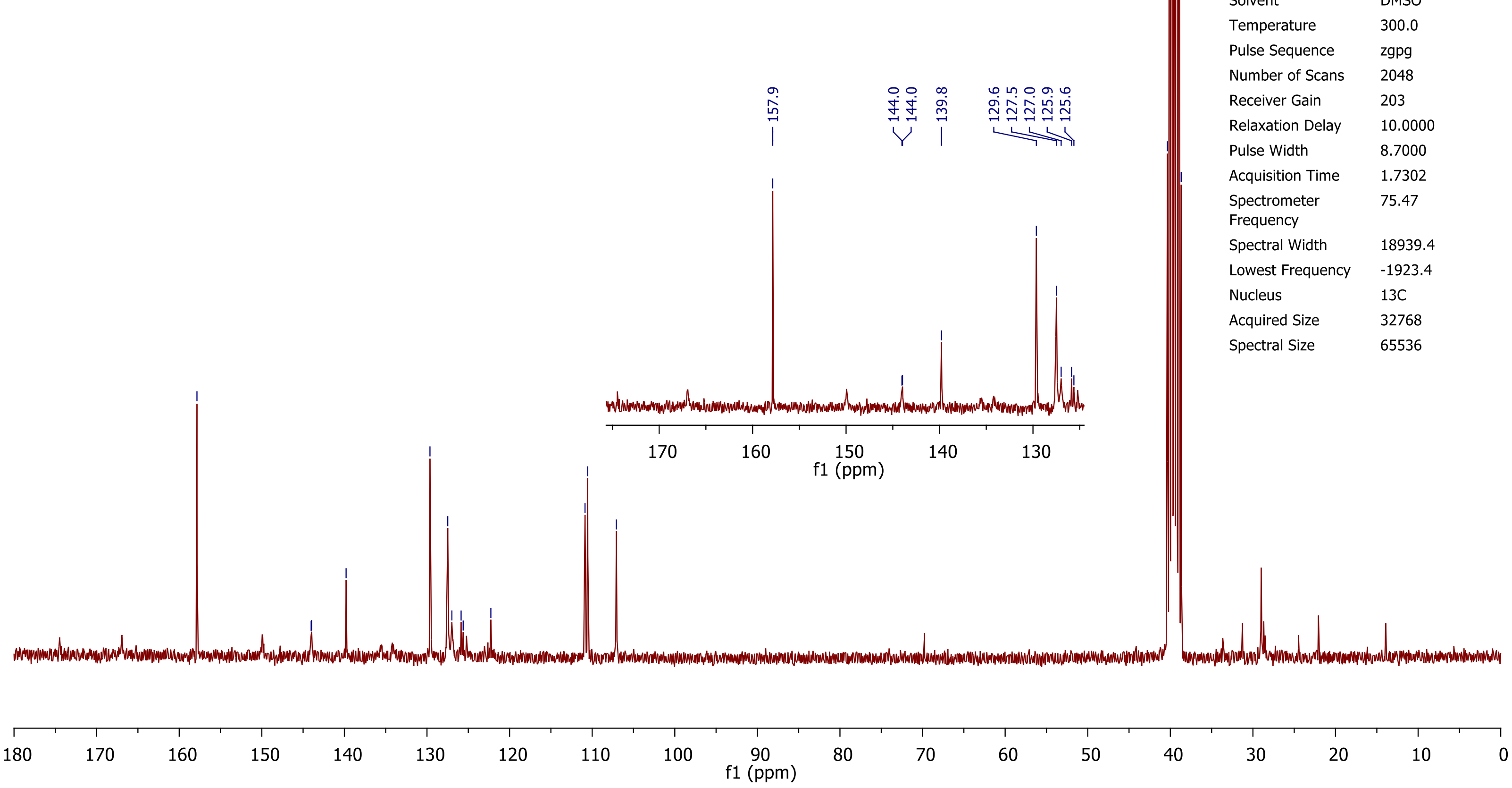

^{19}F NMR of compound **5h**

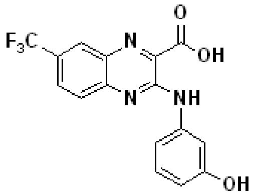

5h

Parameter	Value
Title	BO.208
Origin	Bruker BioSpin GmbH
Spectrometer	spect
Solvent	DMSO
Temperature	300.0
Pulse Sequence	zgfhigqn
Number of Scans	16
Receiver Gain	2050
Relaxation Delay	1.0000
Pulse Width	8.6000
Acquisition Time	0.5768
Spectrometer Frequency	282.40
Spectral Width	113636.4
Lowest Frequency	-85058.4
Nucleus	19F
Acquired Size	65536
Spectral Size	131072

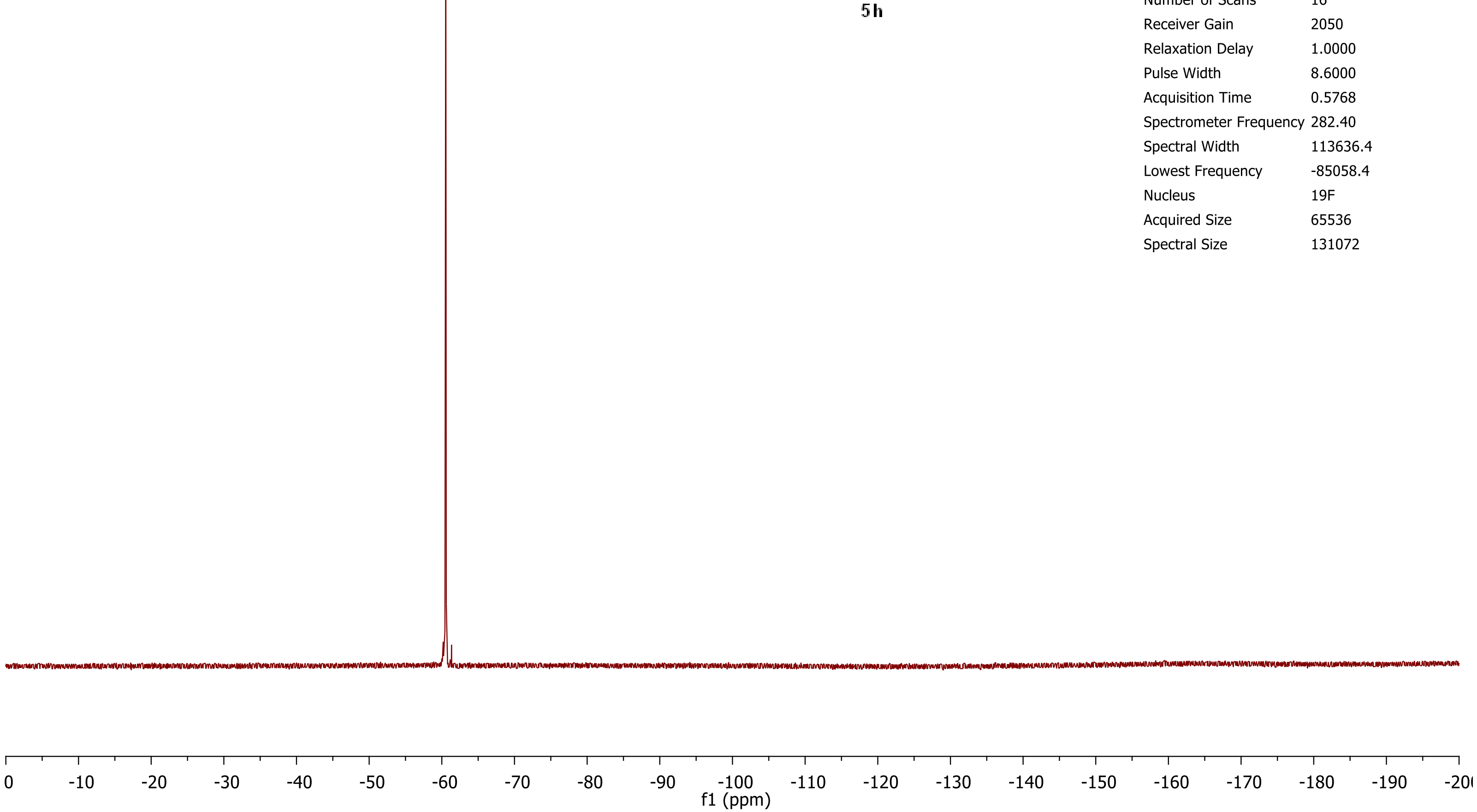

2. Mammalian protein kinase assays

2.1. Inhibition curves of compound **5c**

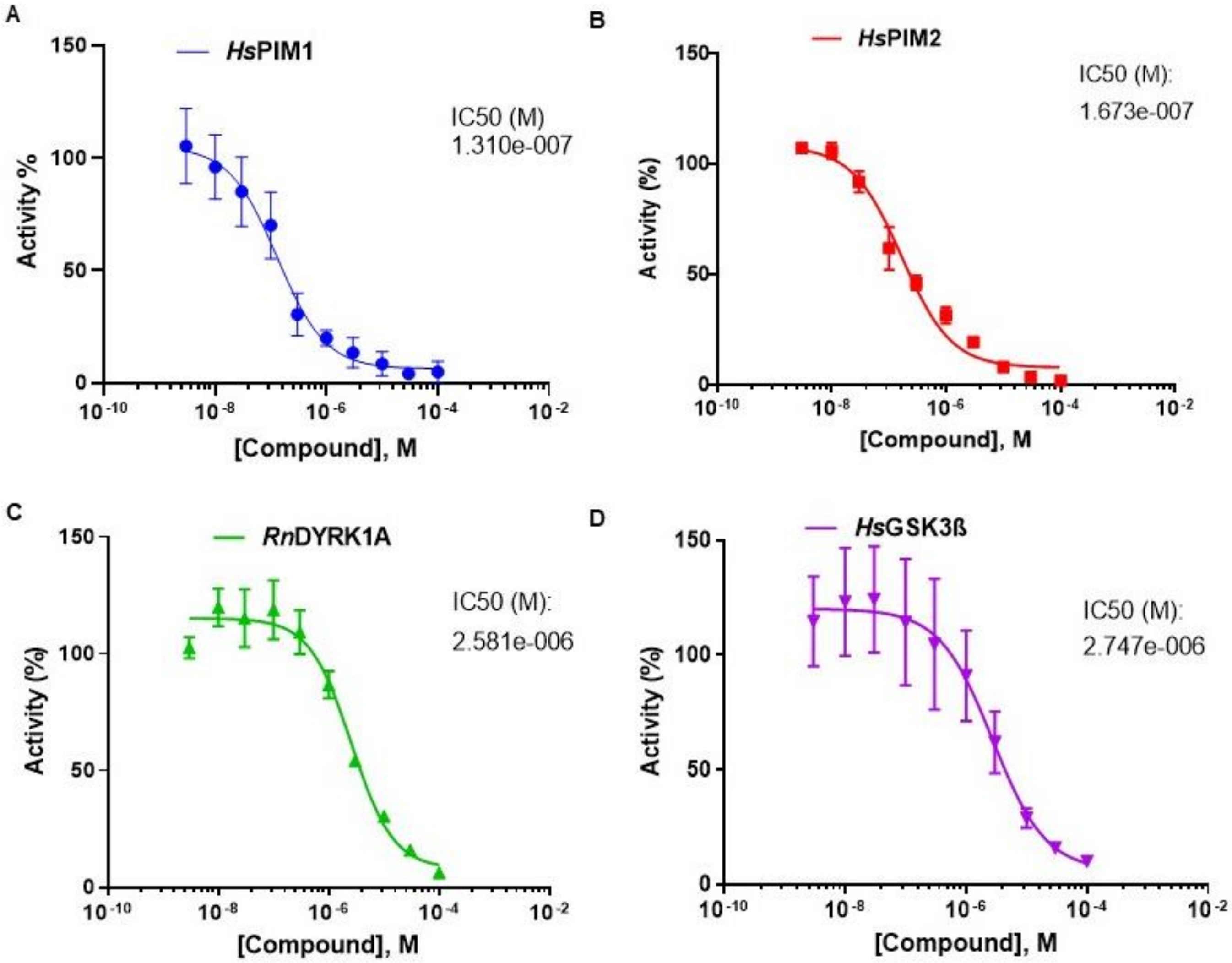

Figure S1. Inhibition curves of compound **5c** on *Hs*Pim1 (A), *Hs*Pim2 (B), *Rn*DYRK1A (C), and *Hs*GSK3β (D).

2.2. Inhibition curves of compound **5e**

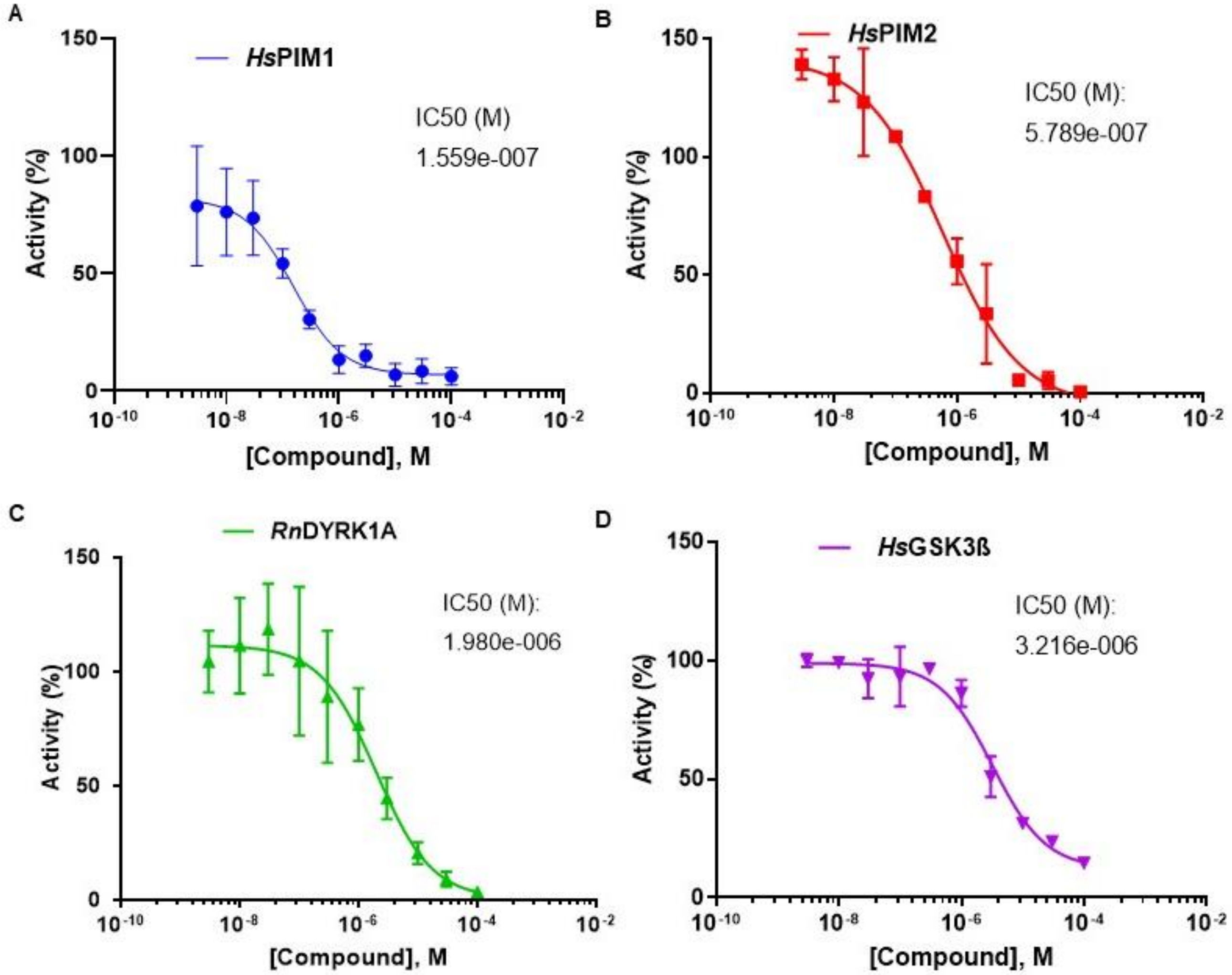

Figure S2. Inhibition curves of compound **5e** on *Hs*Pim1 (A), *Hs*Pim2 (B), *Rn*DYRK1A (C), and *Hs*GSK3β (D).
